# Caspase-8: not so silently deadly

**DOI:** 10.1038/cti.2016.83

**Published:** 2017-01-06

**Authors:** Rebecca Feltham, James E Vince, Kate E Lawlor

**Affiliations:** 1 Inflammation Division, The Walter and Eliza Hall Institute of Medical Research, Parkville, Victoria, Australia; 2 Department of Medical Biology, The University of Melbourne, Parkville, Victoria, Australia

## Abstract

Apoptosis is a caspase-dependent programmed form of cell death, which is commonly believed to be an immunologically silent process, required for mammalian development and maintenance of cellular homoeostasis. In contrast, lytic forms of cell death, such as RIPK3- and MLKL-driven necroptosis, and caspase-1/11-dependent pyroptosis, are postulated to be inflammatory via the release of damage associated molecular patterns (DAMPs). Recently, the function of apoptotic caspase-8 has been extended to the negative regulation of necroptosis, the cleavage of inflammatory interleukin-1β (IL-1β) to its mature bioactive form, either directly or via the NLRP3 inflammasome, and the regulation of cytokine transcriptional responses. In view of these recent advances, human autoinflammatory diseases that are caused by mutations in cell death regulatory machinery are now associated with inappropriate inflammasome activation. In this review, we discuss the emerging crosstalk between cell death and innate immune cell inflammatory signalling, particularly focusing on novel non-apoptotic functions of caspase-8. We also highlight the growing number of autoinflammatory diseases that are associated with enhanced inflammasome function.

## Introduction

**Figure 1:**
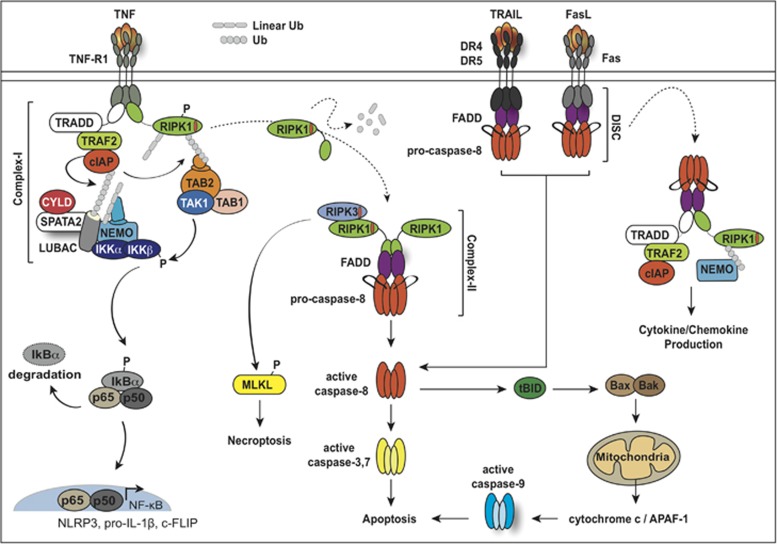
Death receptor signalling pathways. Schematic depicting the activation of the death receptor signalling pathways upon ligation of TNF-TNFR1, FasL-Fas and TRAIL-DR4/DR5. TNF interacts with TNF-R1 and induces timerisation of the receptor. This stimulates recruitment of the adaptor proteins TRADD and RIPK1. TRADD interacts with TRAF2 allowing the recruitment of the cIAP proteins and subsequently LUBAC to the receptor complex. The cIAPs and LUBAC ubiquitylate RIPK1, which stimulates the recruitment and activation of the downstream signalling effectors, culminating in the activation of NF-κB. These ubiquitylation events are important to limit the association of RIPK1 with caspase-8. Similarly, the IKK complex (comprising the kinases IKKα, IKKβ and regulatory subunit NEMO) stimulates the phosphorylation of RIPK1 preventing the association of RIPK1 with caspase-8.^121^ Dysregulation of either the ubiquitylation or phosphorylation of RIPK1 causes the dismantling of complex-I and stimulates the formation and activation of complex-II, which has the ability to drive apoptosis or necroptosis (when caspase activity is inhibited). TRAIL or FasL bind their cognate receptors which induces receptor trimerisation and formation of the death inducing signalling complex (DISC), comprising FADD and caspase-8. Caspase-8 activation can induce death via direct cleavage of downstream caspases, caspase-3/7 in type I cells, or in type II cells caspase-8 can cleave Bcl-2 family member BID to its truncated form that can activate intrinsic apoptosis. Through a less defined mechanism a secondary complex, the ripoptosome, can also form comprising signalling proteins, such as RIPK1, RIPK3 and the cIAPs, which stimulates the upregulation of cytokines and chemokines.

Apoptosis is a genetically encoded process essential for the removal of superfluous or damaged cells. Apoptotic cell death can promote phagocytic clearance of infected cells to limit pathogenic infections,^1, 2^ and is required to delete lymphocytes to prevent autoimmune disease.^3^ Intrinsic ‘mitochondria-dependent' apoptosis is triggered by cellular stressors (for example, growth factor withdrawal) and is tightly regulated by the pro- and anti-apoptotic members of the BCL-2 protein family (reviewed in Delbridge *et al.*^4^). In contrast, the extrinsic ‘death receptor-mediated' apoptotic pathway is induced by ligand binding, which via a series of events can activate the death receptor complex or ripoptosome that activates caspase-8 (Figure 1). Both pathways merge following initiator caspase activation to trigger the activation of effector caspases, caspase-3 and caspase-7, resulting in ordered cellular breakdown.

Death receptor ligation not only induces caspase-8-mediated apoptosis but also results in transcription factor activation (Figure 1). Pro-inflammatory cytokines, such as tumour necrosis factor (TNF), are potent activators of NF-κB, regulating the transcription of a variety of inflammatory genes, including TNF itself. In fact, enhanced TNF production and TNF receptor 1 (TNFR1) signalling is associated with macrophage accumulation and inflammatory cytokine production in common autoimmune pathologies, such as Crohn's disease, rheumatoid arthritis and psoriasis. Although biologics targeting TNF have proven relatively efficacious in the treatment of these common diseases, understanding the pathways that regulate TNFR1 signalling is key to identifying next-generation therapies. This is particularly true in view of the crosstalk, and shared signalling components, of cell death and pattern recognition receptor (PRR)-mediated innate immune signalling pathways. Similar to the death receptors, Toll-like receptors (TLRs) detect pathogen molecules and host-derived damage-associated molecular patterns (DAMPs) to induce the transcription of death ligands, including TNF and directly trigger caspase-8-dependent apoptosis and caspase-independent necroptotic cell death. TLR signalling may therefore contribute to the pathogenic effects of TNF in autoimmune diseases, such as arthritis.

In this review, we give an overview of innate immune and cell death signalling pathways, particularly focusing on pathways that signal to caspase-8. We outline non-apoptotic functions for caspase-8, including its ability to repress necroptosis, regulate cytokine transcription, and interact with inflammasomes to direct their signalling. Importantly we describe emerging evidence suggesting that in specific circumstances caspase-8 activity determines how pro-inflammatory interleukin-1β (IL-1β) processing and activation occurs. Finally, we highlight the increasing number of autoinflammatory diseases linked to mutations in cell death machinery, which can lead to excessive inflammasome activation.

## Death receptor signalling

The TNF superfamily comprises a number of type I plasma membrane proteins that feature a common cysteine rich extracellular-ligand binding domain, a membrane-spanning region and a C-terminal intracellular tail. Notably, only a subset of these receptors also harbour a death domain (DD) that can directly induce apoptosis. Of these, TNFR1 (p55), TNF-related apoptosis-inducing ligand receptor 1 (TRAIL-R1/DR4) and 2 (TRAIL-R2/DR5) and Fas (CD95/APO-1) are the most widely studied in response to their respective ligands TNF/lymphotoxin-α, TRAIL and Fas ligand (FasL; Figure 1).

### TNFR1 signalling, ripoptosome formation and activation of caspase-8

TNF is expressed predominantly by activated macrophages and T lymphocytes, as a 26 kDa protein on the plasma membrane. Cleavage of transmembrane TNF by the metalloproteinase, TACE (TNF-α-converting enzyme), results in the release of a 17 kDa soluble form.^5^ Despite many studies focusing specifically on the soluble form of TNF, both membrane bound (mTNF) and soluble TNF (sTNF) are biologically active and accumulating evidence suggests that each form of TNF have distinct roles in inflammatory responses. For example, anti-human TNF monoclonal antibodies (infliximab and adalimumab) and soluble human TNFRII (etanercept) are highly efficacious in neutralising sTNF to treat rheumatoid arthritis. In contrast, anti-TNF biologicals, particularly etanercept fail in Crohn's disease possibly due to ineffective targeting of mTNF that drives localised pathology.^5, 6^

Binding of TNF to TNFR1 results in the formation of a pro-survival TNF receptor signalling complex (TNFRSC; also known as complex-I), comprising the core proteins, TNFR1-associated death domain protein (TRADD), TNF receptor-associated factor 2 (TRAF2), Receptor-Interacting Protein Kinase 1 (RIPK1), cellular inhibitor of apoptosis proteins (cIAP1/cIAP2) and the linear ubiquitin chain assembly complex (LUBAC, a hetero-trimeric complex comprising SHARPIN, HOIL-1 and HOIP) (Figure 1). Conformational changes in TNFR1 facilitate the intracellular recruitment of TRADD via common death domains (DD), which allows interactions between TRADD, TNFR1 and RIPK1.^7, 8, 9, 10^ TRAF2 associates directly with TRADD via a TRAF binding domain and acts as an adaptor for the recruitment of the E3 ubiquitin ligases, cIAP1 and cIAP2, via a cIAP1/2-interacting motif (CIM).^11^ The cIAPs are arguably the most important E3 ubiquitin ligases recruited to the TNFRSC, where they function to recruit LUBAC (Figure 1). Subsequently, the cIAPs and LUBAC ubiquitylate NEMO and RIPK1 to promote cell survival through TAK1- and IKK-dependent transcriptional activation of canonical NF-κB, resulting in cytokine (for example, TNF and IL-6) and pro-survival protein (for example, c-FLIP and cIAPs) induction.^12^

The activation of NF-κB only constitutes one arm of the TNFRSC. When TNFR1 induced pro-survival responses are compromised (loss of the IAPs or inhibition of TAK1), complex-I dissociates from the receptor, and RIPK1 together with TRADD associate with the adaptor protein FADD (Fas-associated protein with death domain) and pro-caspase-8 to form complex-II (Figure 1).^7^ This complex has the ability to drive apoptosis through the dimerisation and auto-activation of caspase-8, resulting in cleavage and activation of downstream effector caspases, caspase-3 and caspase-7. Formation of this secondary complex is tightly regulated by the inducible caspase-8 inhibitor c-FLIP (cellular FLICE-inhibitor protein), which dictates the activity of caspase-8 and determines if apoptosis ensues.^13^ If caspase-8 activity is compromised, TNF stimulation under appropriate circumstances, such as IAP depletion, triggers necroptosis (Figure 1). TNF-induced necroptosis requires the kinase activity of RIPK1 and RIPK3, and the RIPK3 substrate, the pseudokinase Mixed Lineage Kinase domain-Like protein (MLKL). The phosphorylation of MLKL by RIPK3 activates MLKL, and is thought to result in a conformational change that leads to the exposure of the N-terminal four-helix bundle (4HB) killing domain.^14, 15, 16^ In its oligomerised form, MLKL migrates to plasma membranes, where it is purported to cause death via pore formation,^17^ thus leading to inflammatory DAMP release (for example, IL-1α and HMGB1).

In contrast to the cIAPs, X-linked IAP (XIAP) has not yet been detected in the TNFRSC, but is widely reported to bind and directly inhibit apoptotic caspases, caspase-3, -7 and -9. Recently, however, XIAP has also been shown to critically regulate both cell death and innate immune responses following TNF and TLR ligation (discussed later in this review). Despite this fact, no major perturbation of TNF-induced canonical NF-κB, p38 or JNK signalling pathways has been reported in XIAP-deficient dendritic cells.^18^ Nevertheless, inhibition of both the cIAPs and XIAP with compounds that mimic the natural inhibitor of IAPs, Smac/DIABLO, termed ‘Smac mimetics', or the genetic loss of all three IAPs, leads to the formation of a complex akin to complex-II, termed the ‘ripoptosome', comprising RIPK1, RIPK3, FADD, caspase-8 and cFLIP.^19, 20^ This complex forms without the need for receptor ligation and functions to drive caspase-8-mediated apoptosis or caspase-independent necroptosis.

### TRAIL- and FAS-mediated activation of caspase-8

Distinct from TNFR1, FAS and TRAIL-RI and -RII signalling culminates from the formation of a death inducing signalling complex (DISC) at the receptor cytoplasmic tail (Figure 1). This DISC complex is remarkably similar to the TNFR complex-II, and the ripoptosome, in that it forms from a DD interaction between the receptor and FADD, which is followed by death effector domain (DED)-mediated recruitment, oligomerisation and activation of caspase-8 or -10. FasL and TRAIL receptors are considered classic examples of ‘death receptors', as their primary function is to drive caspase activation and apoptosis, unlike TNFR1 whose primary role is to activate NF-κB to drive inflammatory gene transcription. However, recently it has been appreciated that FasL and TRAIL receptor engagement can lead to pro-inflammatory cytokine and chemokine expression.^21, 22, 23^ Not surprisingly, this inflammatory programme is somewhat dependent on RIPK1, but more critically dependent on caspase-8.^21^ A scaffolding function for caspase-8 seems likely, as inhibiting caspase activity does not suppress Fas-mediated cytokine/chemokine expression or function.^21^ Similar to complex-II and the ripoptosome, cFLIP is also recruited to the DISC to determine the cellular fate. Although cFLIP_s_ acts as a direct inhibitor of caspase-8, cFLIP_L_ is incorporated into the DISC thereby preventing pro-caspase-8 interdomain processing but facilitating non-apoptotic caspase-8 cleavage of a limited number of substrates around the DISC.^24, 25^

Like TNFR1, FAS and TRAIL-R signalling events are important for regulating pathogen clearance and immune responses, as well as for driving inflammatory disease via effects on cell viability and/or pro-inflammatory cytokine and chemokine induction.^26^ The critical role of Fas/FasL signalling in disease is highlighted by the autoimmune lymphoproliferative syndrome (ALPS) that occurs in patients harbouring Fas/FasL mutations, and C57BL/6.*Fas*^*lpr*^ and C57BL/6.*FasL*^*gld*^ mice, respectively.^3^

## Crosstalk between TLR and TNFR1 signalling pathways: non-apoptotic activities for caspase-8

PRRs, including TLRs and inflammasome-forming NOD-like receptors (NLRs), act as the major sensors for invading pathogens and, like TNFR1, have an essential role in coordinating the innate immune response to clear microbial infections. Recent work has revealed significant crosstalk between TNFR1 and TLR signalling pathways, where RIPK1 and RIPK3 interact with TIR-domain-containing adaptor-inducing interferon-β (TRIF) via common RIP Homotypic Interacting Motif domains (RHIM) to induce cell death signalling.^27, 28^ Work has also highlighted that caspase-8 is not simply the initiator caspase for cell death but is a key player in regulating inflammatory responses.

### Caspase-8-mediated repression of necroptosis

The best recognised non-apoptotic functions for FADD and caspase-8 is the repression of necroptotic signalling, where inhibition of caspase-8 by pathogen/mammalian inhibitors (for example, CrmA, vICA and cFLIPs),^29^ chemical inhibition (for example, ZVAD-fmk),^28, 30^ or genetic loss,^31^ promotes necroptosis upon death receptor or TLR signalling. The seminal studies performed by the laboratories of Mocarski and Green, and more recently Strasser, highlighted this fact when they rescued the embryonic lethality of caspase-8 knockout mice (embryonic day 10.5) by co-deletion of the necroptotic regulator *Ripk3* or *Mlkl* itself (Table 1).^32, 33, 34^ *Ripk3*^−/−^*Caspase-8*^−/−^ and *Mlkl*^−/−^*Caspase-8*^−/−^ mice are born viable and healthy, although similar to mice lacking Fas/FasL they succumb to SLE-like lymphoproliferative disease.^32, 33, 34, 35^ Not surprisingly, mice deficient in the caspase-8 adaptor FADD,^36, 37^ or c-FLIP (*Cflar* gene)^38, 39^ are also embryonic lethal. However, only *Fadd*^−/−^ mice are rescued by blockade of necroptotic activity via deletion of *Ripk3* or *Mlkl*,^34, 39, 40^ suggesting that in the absence of c-FLIP, FADD-caspase-8 homodimers induce lethal apoptotic signalling. Consistent with this, loss of *Fadd* in the *Cflar*^−/−^*Ripk3*^−/−^ mouse restores viability.^39^ Interestingly, early embryonic lethality of *Fadd*^−/−^ and/or *caspase-8*^−/−^ mice is driven by TNFR1 signalling, as RIPK1 or TNFR1 deficiency but not TRIF loss prolongs survival, although mice still exhibit perinatal lethality akin to *Ripk1*^−/−^ mice (Table 1).^28, 41, 42, 43^ The lethality in *Ripk1*-deficient mice is partially rescued by loss of MyD88, TRIF and/or TNFR1.^41, 43^ More importantly, loss of RIPK3 or MLKL also delays death, demonstrating a repressive role of RIPK1 on inflammatory necroptotic signalling.^41, 43^ Intriguingly, complete rescue of *Ripk1*^−/−^ mice was only achieved by elimination of both necroptotic (that is, RIPK3) and apoptotic (that is, caspase-8 or FADD) machinery.^41, 42, 43^

Caspase-8 has also been shown to repress necroptosis in a cell and tissue-specific manner. In gut epithelial cells, caspase-8 maintains the gut integrity to microbial challenge by preventing lethal necroptotic cell death and epithelial cell shedding.^44^ Likewise, caspase-8 expression in the skin prevents inflammatory necroptotic cell death.^45^ In the haematopoietic system caspase-8 apoptotic activity is widely thought to restrict lymphocyte accumulation, where ALPS develops in caspase-8-deficient or Fas-deficient patients, as well as in T cell-specific caspase-8 knockout mice, Fas-, FasL- or membrane FasL-deficient mice.^3, 46^ However, unexpectedly, NF-κB1 signalling has recently been shown to drive the lymphoproliferation in mice deficient in Fas.^47^ Necroptosis has been suggested to contribute to the contraction of T cell responses, as cell death in caspase-8 deficient T cells upon T cell receptor ligation is prevented by RIPK1 kinase inhibition or RIPK3 co-deletion.^48^ However, recent reports in MLKL and caspase-8 deficient mice show subtle differences in a discrete subset of inflammatory genes, suggesting closer examination of non-necroptotic activities of RIPK3 and RIPK1 is warranted.^34^ Caspase-8 loss in mature myeloid cells (that is, dendritic cells, macrophages and neutrophils) also does not lead to spontaneous or TLR-induced necroptotic cell death,^49^ however, reduced levels of IAPs renders cells permissive to necroptosis.^50^

How caspase-8 prevents necroptotic signalling is not fully understood, although the catalytic activity of caspase-8, rather than its autocatalytic processing, appears to be essential to block necroptosis.^32^ Caspase-8-mediated cleavage of RIPK1 and RIPK3 is widely believed to limit necroptotic signalling, where necroptosis activation requires oligomerisation of full length RIPK1 and RIPK3 via RHIM-RHIM interactions to form an amyloid-like fibril structure.^51^ Indeed, overexpression of mutant RIPK1 and RIPK3 RHIMs blocked necroptosis, however, these RHIM mutations may also inhibit upstream RIPK1/3 kinase activity. Nevertheless, increased phosphorylated MLKL activity is associated with reduced caspase-8 levels and amyloid-like RIPK1 and RIPK3 deposits in the cortical lesions found in the brains of multiple sclerosis patients.^52^

The levels of the different cFLIP isoforms, and the interaction of these isoforms with caspase-8 at the DISC, have also been suggested to dictate ripoptosome stability, and thus influence apoptotic and necroptotic death signalling. For instance, low cFLIP_L_ levels trigger caspase-8 oligomerisation and activation to induce apoptosis and inhibit necroptosis, whilst high levels block apoptotic activity. In the case of cFLIP_s,_ caspase-8-c-FLIP_s_ complexes inhibit apoptosis and induce necroptosis.^53^ Finally, caspase-8 also cleaves the deubiquitylase CYLD (cylindromatosis), which is required to deubiquitylate and activate RIPK1-mediated necroptosis in response to TNF,^54^ thus potentially providing an alternative or additional mechanism for silencing necroptosis. Recently, TLR4-TRIF-caspase-8 signalling was also shown to cleave CYLD to limit autocrine TNF-driven, type 1 IFN-dependent necroptosis in macrophages.^55^ Intriguingly, this work contrasts a similar study that suggested type 1 IFN induces necroptosis in the absence of TNFR1/2.^56^

### Transcriptional role for caspase-8 in cytokine production

In response to TNFR and TLR ligation cytokine transcription can be activated in a RIPK1 and RIPK3 dependent manner.^57, 58, 59^ Subsequent studies have sought to discern if caspase-8 also regulates transcription of inflammatory genes. Cuda *et al.* recently documented that deletion of caspase-8 in dendritic cells (*Caspase-8*^*CD11cCre*^) is associated with heightened inflammatory cytokine and chemokine levels and autoimmune SLE-like disease, which is driven by RIPK1 and MyD88, but not RIPK3.^60^ Myeloid-specific loss of caspase-8 (*Caspase-8*^*LysMCre*^) also led to mild autoimmunity, albeit with a less prominent inflammatory cytokine/chemokine profile that could be rescued by RIPK3 co-deletion.^61^ In line with *in vivo* findings, caspase-8 deficient macrophages exhibited altered transcriptional responses to TLR ligation *in vitro*, where enhanced TLR4-induced TNF and IL-6 production was reduced by either necrostatin-1 or RIPK3 loss.^61^ Unexpectedly, *in vivo* cytokine responses to LPS were blunted in *Caspase-8*^*LysMCre*^ mice,^61^ akin to defective cytokine production reported in *Ripk3*^−/−^*Caspase-8*^−/−^*, Mlkl*^−/−^*Fadd*^−/−^ and *Ripk3*^−/−^*Fadd*^−/−^ mice, and macrophages exposed to TLR3/4 stimuli or Gram-negative bacteria (for example, *Citrobacter rodentium*).^40, 58, 62^ Results therefore suggest that caspase-8 is required for optimal TLR-induced cytokine transcriptional responses. However, the fact there are conflicting reports as to whether defective canonical NF-κB activation is responsible for reduced cytokine production in *Ripk3*^−/−^*Fadd*^−/−^, *Mlkl*^−/−^*Fadd*^−/−^ and *Ripk3*^−/−^*Caspase-8*^−/−^ macrophages to TLR ligation,^40, 58, 62, 63^ suggests that further studies are warranted. Globally, results point to an important role for caspase-8 levels in dictating TLR-induced transcriptional responses in a cell-by-cell manner.

## Caspase-8 regulation of the inflammasome and IL-1β activation

### Inflammasome activation

**Figure 2:**
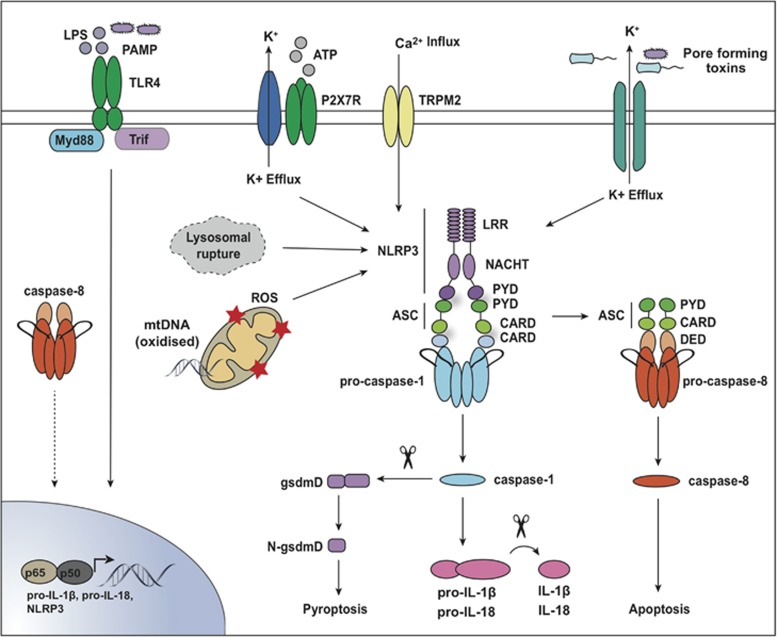
Models for canonical inflammasome activation. Activation of the canonical NLRP3 inflammasome requires a priming step to induce transcription of inflammasome machinery, namely pro-IL-1β and NLRP3. This priming step can be initiated via caspase-8, although this is not well characterised. Upon sensing a range of DAMPS, PAMPS or environmental irritants, NLRP3 oligomerises via its NACHT domain, subsequently the N-terminal pyrin domain (PYD) facilitates homotypic interactions with the PYD domain of ASC. ASC oligomerisation into prion-like fibril structures, visualised as discrete specks, can interact with caspase-1 via common CARD domains to facilitate caspase-1 proximity-associated activation. Active caspase-1 cleaves pro-IL-1β and pro-IL-18 to their mature bioactive forms, and induces gasdermin D-mediated pyroptosis. The exact mechanism for NLRP3 activation remains contentious, but potassium (K^+^) efflux is widely believed to be the common trigger. Other potential triggers include calcium (Ca^2+^) influx, lysosomal rupture, ROS production and mitochondrial cell death (e.g., oxidised DNA, ROS, cardiolipin). Of note, under certain circumstances ASC can interact via its PYD domain with the DED of caspase-8 leading to its oligomerisation and activation to induce apoptosis and cleave IL-1β.

Inflammasomes are large multimeric protein complexes that typically comprise a NOD-like receptor (NLR), the adaptor ASC (apoptosis-associated speck-like proteins containing a CARD, encoded by the *PYCARD* gene) and caspase-1 (Figure 2). The primary function of inflammasomes is to activate caspase-1 to cleave precursor IL-1β and IL-18 into their mature bioactive forms. However, a physiological role for the lytic form of cell death, pyroptosis, which ensues following inflammasome activation has also been described. To date there are at least 6 NLR proteins suggested to form inflammasomes, NLRP1, NLRP3, NAIP/NLRC4 (IPAF), NLRP6, NLRP7, NLRP12, as well as non-NLR inflammasomes, the HIN 200 family members AIM (Absent in melanoma)-like receptors, AIM2 and IFI-16 (Interferon-gamma inducible protein-16), and the tripartite motif-containing family member Pyrin.^64^ In addition to these canonical inflammasomes, caspase-11 (human orthologues caspase-4 and caspase-5) has recently been revealed to be the cytosolic receptor for intracellular LPS derived from Gram-negative bacteria (for example, *Burkholderia spp.* and *Eschericia coli*.).^65, 66, 67^ Termed the noncanonical inflammasome, LPS derived from Gram-negative bacteria induces caspase-11 activation. Caspase-11, like caspase-1, cleaves gasdermin D to cause pore formation and pyroptotic cell death. However, caspase-11 also activates the NLRP3 inflammasome indirectly, reportedly as a result of gasdermin D-mediated potassium (K^+^) efflux.^67, 68^

Canonical inflammasome activation is generally thought of as a two-step model. TLR/TNFR ligation provides the first signal, termed inflammasome priming, which is required for the transcriptional upregulation of inflammasome machinery (for example, NLRP3) and pro-IL-1β. The second signal, the trigger, such as a PAMP (Pathogen associated molecular pattern) or DAMP, is then required to induce inflammasome assembly and activation in the cytosol (Figure 2). A number of inflammasomes have well-defined pathogen triggers that implicate them in innate immune responses to infection. NLRP1 senses lethal toxin from *Bacillus anthracis*, AIM2 senses cytosolic DNA, and pyrin is triggered by Rho GTPase inactivating bacterial toxins/effectors, such as *Clostridium difficile* toxin A/B.^69, 70^ In contrast, the most widely studied inflammasome, NLRP3, is triggered by a diverse range of PAMPs (for example, *Streptococcus pneumoniae* and *Staphylococcus aureus*), host-derived DAMPs (for example, monosodium urate crystals, islet amyloid polypeptide and ATP), and environmental irritants (for example, silica and alum).^70^ These diverse triggers implicate the NLRP3 inflammasome in the pathogenesis of a variety of inflammatory and autoimmune diseases, including type 1 diabetes, gout, and silicosis.^70^ Yet the importance of tight regulation of the NLRP3 inflammasome and IL-1β is best reflected by the potentially lethal autoinflammatory diseases, termed cryopyrin associated periodic syndromes (CAPS), which occur in humans and mice harbouring various activating mutations in *NLRP3*.^71^

Based on the diverse stimuli that trigger the NLRP3 inflammasome one might predict a unifying activation mechanism exists. Gabriel Nunez's laboratory proposed K^+^ efflux as the universal trigger for NLRP3.^72^ However, recent reports suggest that K^+^ efflux is not required for NLRP3 inflammasome activation triggered by peptidoglycan-N-acetylglucosamine-induced hexokinase release from the mitochondrial outer membrane,^73^ or for caspase-8-mediated activation of the NLRP3 inflammasome in human monocytes.^74^ Other common models for NLRP3 activation include phagolysosomal rupture and cathepsin release, ion channel flux and calcium influx, and reduced cyclic AMP. Activation of the mitochondrial cell death pathway is also postulated to trigger canonical NLRP3, via mitochondrial reactive oxygen species (ROS), oxidised mitochondrial DNA release, or direct NLRP3 binding by the mitochondrial membrane lipid cardiolipin.^75, 76^ However, recent studies have genetically disputed the role of the mitochondria and associated proteins in canonical NLRP3 activation.^58, 72, 75^ Hence, the elusive common NLRP3 activating mechanism remains of outstanding interest.

### Caspase-8-mediated IL-1β activation

**Figure 3:**
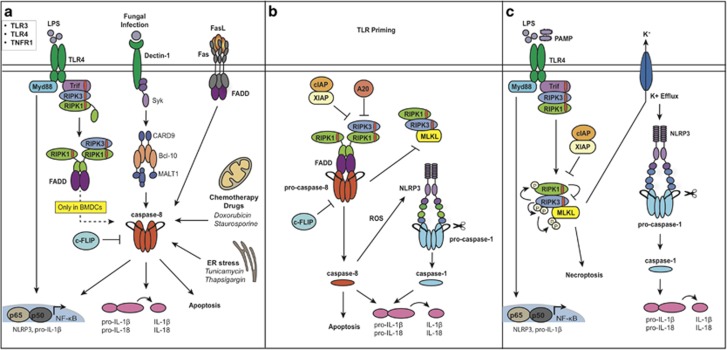
Novel roles for caspase-8 in IL-1β regulation. Following exposure to TLR/TNFR stimuli to induce transcription of pro-IL-1β (and NLRP3) caspase-8 levels can regulate IL-1β activity. (**a**) Caspase-8 can be activated by a wide variety of stimuli to directly cleave IL-1β, including TLR4 or Fas death receptor ligation, fungal protein binding of dectin-1 that induces a CARD9-Bcl-10-MALT1 complex to trigger activation, as well as apoptosis-inducing stimuli. Of note, reports illustrating that TLR4 stimulation can directly stimulate caspase-8-mediated cleavage of IL-1β are restricted to bone marrow derived dendritic cells (BMDCs). (**b**) When IAPs are inhibited/genetically removed (or A20 is absent), the ripoptosome forms and caspase-8 can either directly cleave IL-1 and/or trigger the NLRP3 inflammasome, whereby caspase-1 cleaves IL-1β. In this scenario caspase-8 causes apoptosis and limits necroptosis, possibly by cleavage of RIPK1 and RIPK3. (**c**) When caspase-8 levels are low/absent and IAPs are inhibited/genetically removed, the necrosome is formed and MLKL is activated through RIPK3 kinase activity to trigger necroptosis and NLRP3 inflammasome, possibly via K^+^ efflux.

Interest in caspase-8 as an inflammatory caspase stemmed from a study showing that TLR3 or TLR4 ligation and protein synthesis inhibition resulted in caspase-8 dependent activation and cleavage of precursor IL-1β at the same site as caspase-1.^77, 78^ Subsequently, a range of stimuli have been shown to activate caspase-8 to cleave IL-1β (and IL-18) in TLR or TNF primed cells (Figure 3a), where TLR4 ligation alone can trigger spontaneous caspase-8-induced IL-1β activation in bone marrow derived dendritic cells (BMDCs).^79^ Other stimuli include natural death receptors Fas^80^ and DR3,^81^ bacterial and fungal c-type lectin receptor, dectin-1,^82^ as well as chemicals that induce ER stress (for example, tunicamycin)^83^ or chemotherapeutic compounds, such as doxorubicin, staurosporine^84^ and histone deacetylase inhibitors (Figure 3a).^85^ Fas-mediated cleavage of IL-1β, draws parallels to the TLR-mediated pathway in dendritic cells, as it too is dependent on FADD and caspase-8 and occurs independent of the NLRP3 inflammasome, however, it does not require RIPK3.^80^ Interestingly, while Dectin-1 signalling can activate caspase-8 to cleave IL-1β (Figure 3a),^82^ other studies suggest that dectin receptors also utilise the canonical NLRP3 inflammasome.^86, 87^ How chemical stressors trigger apoptosis, caspase-8 and thus IL-1β activation is unclear, but is likely to involve changes in expression of cell death inhibitory components.^84^ In this regard, recent work shows that in LPS- or TNF- primed murine macrophages and dendritic cells, genetic or chemical loss of all three IAP proteins, and pivotally XIAP, can promote caspase-8-mediated IL-1β activation (Figure 3b).^18, 50, 78^ Furthermore, loss of c-FLIP enhances caspase-8 activation of IL-1β secretion upon treatment with Smac mimetic, FasL or heat-killed *Candida albicans* (Figures 3a and b).^88^

### Caspase-8 and the activation of the NLRP3 inflammasome

A number of recent studies have also suggested that caspase-8 can activate NLRP3 (Figure 3b). For example, upon chemical or genetic loss of IAP activity in LPS-primed murine macrophages and dendritic cells, TLR-TRIF-RIPK1-RIPK3-caspase-8 signalling has been shown to not only directly cleave IL-1β, but to also induce NLRP3 inflammasome activation,^18, 50, 78^ independent of the kinase activity of RIPK1 and RIPK3.^50^ In contrast, Gurung *et al.* suggested that FADD-RIPK3-caspase-8 associates with the NLRP3 inflammasome to promote canonical (for example, ATP and nigericin) and non-canonical caspase-11 (for example, *C. rodentium*) inflammasome activation.^62^ However, as other groups have reported relatively normal canonical NLRP3 inflammasome activation (for example, ASC oligomerisation and Caspase-1 cleavage) in *Ripk3*^−/−^*Caspase-8*^−/−^ (and *Caspase-8*^−/−^) myeloid cells upon sufficient priming signals, this finding warrants re-examination.^49, 50, 89^ A further model proposed in response to *Yersinia* infection is that a RIPK1-FADD-caspase-8 complex can directly cleave caspase-1 in the absence of NLRP3 and NLRC4.^63, 90^ Despite these variations, we recently established an upstream position for caspase-8 in ripoptosome-mediated, but not canonical, NLRP3 inflammasome activation.^50^ In this study, to avoid issues in defective priming in *Ripk3*^−/−^*Caspase-8*^−/−^ macrophages, NLRP3 was triggered in unprimed macrophages (which express low levels of NLRP3) and caspase-1 cleavage assessed as an activation measurement. Upon Smac mimetic-induced inhibition of IAPs, caspase-1 activity was blocked in *Ripk3*^−/−^*Caspase-8*^−/−^ and *Nlrp3*^−/−^ macrophages. In contrast, in response to canonical NLRP3 stimulus, nigericin, caspase-1 cleavage was only blocked in the absence of NLRP3 demonstrating that caspase-8 is not required for canonical inflammasome activation.^50^

In bone marrow derived dendritic cells a TLR-TRIF-RIPK3 platform not only activates caspase-8 to directly cleave IL-1β, but also triggers NLRP3 inflammasome activation.^79^ Intriguingly this IL-1β activation occurs largely in the absence of cell death, and independent of the kinase activities of RIPK1 and RIPK3. However, unexpectedly, a RIPK3 kinase inhibitor, which blocks necroptotic activity and amplifies caspase-8 activity,^91^ actually heightened IL-1β activation.^79^ An alternative TLR-TRIF-RIPK1-FADD-caspase-8-mediated, K^+^ independent, route to NLRP3 inflammasome activation was also recently described in human BlaER1 monocytes.^74^ Of note, ROS activity was suggested to be involved in ripoptosome-triggered NLRP3 activation upon IAP loss in TLR-primed macrophages.^78^ Further studies are, therefore, needed to address how RIPK1-RIPK3-FADD-caspase-8 complexes can trigger the NLRP3 inflammasome.

Recent work has also documented that when caspase-8 levels are reduced TLR ligation can also trigger NLRP3 inflammasome activation via necroptotic activity (Figure 3c). Caspase-8 deficiency in dendritic cells sensitised mice to LPS-induced lethality through RIPK3-driven NLRP3 inflammasome activation.^49^ Subsequently, TLR2/4 ligation in caspase-8 deficient murine bone marrow derived dendritic cells and macrophages, as well as human BalER1 monocytes, was shown to trigger necroptotic activation of the NLRP3 inflammasome, in a RIPK1 and RIPK3 kinase-dependent manner.^49, 50, 74^ Likewise, in TLR-primed macrophages IAP loss and inhibition/genetic loss of caspase-8 leads to RIPK3 kinase-dependent MLKL-driven activation of NLRP3.^50^ It has been proposed that following TLR3 pathway activation, catalytically inactive caspase-8 (that is, by chemical inhibition) acts as a scaffold to recruit a FADD-RIPK1-RIPK3 complex that subsequently, via the kinase activity of RIPK3, triggers MLKL-dependent NLRP3 inflammasome activation.^92^ Supporting this idea is the fact that TLR3-induced necroptotic activation of the NLRP3 inflammasome was not restored by complementing RIPK3 into *Ripk3*^−/−^*Caspase-8*^−/−^ macrophages.^92^ However, defective TLR3/4 induced inflammasome priming in *Ripk3*^−/−^*Caspase-8*^−/−^ macrophages may complicate these findings.^58^ Furthermore, the fact TLR-induced RIPK3-MLKL can trigger NLRP3 inflammasome activation upon genetic loss of caspase-8, or RIPK1, also suggests that a scaffolding function for caspase-8 and RIPK1 may only be relevant to dsRNA.^43, 49, 50, 92^

### Inflammasome-induced caspase-8 activation and apoptosis

Biochemically and structurally, caspase-8 has been shown to interact via its DED domain with the PYD domain of inflammasome adaptor ASC, thereby making it plausible that multiple ASC-containing inflammasomes utilise caspase-8 signalling.^93, 94^ Studies have shown that in the absence of caspase-1, canonical NLRP3 (for example, nigericin) and AIM2 (for example, *Francisella tularensis* and DNA) inflammasome activation can cause ASC-induced caspase-8 oligomerisation into filamentous structures capable of inducing apoptosis.^93, 94, 95^ Furthermore, in the absence of caspase-1/11, canonical NLRP3-ASC activation of caspase-8 induces not only apoptosis but also IL-1β activation in dendritic cells, albeit with delayed kinetics compared with caspase-1.^89^ Notably, ASC-caspase-8 triggered apoptosis may occur preferentially in wild type dendritic cells exposed to low concentrations of inflammasome stimuli.^94^ Perhaps in this scenario, apoptotic cell death would be a preferable immunologically silent route for the cells demise. The strongest physiological evidence for an ASC-caspase-8 containing inflammasome is the fact that *Francisella tularensis* activates a caspase-1 independent AIM2-ASC inflammasome to drive IL-18-dependent IFN-γ production.^96^ In the case of *Salmonella* infection, an NLRC4-ASC-caspase-8-caspase-1 inflammasome has also been shown to induce pro-IL-1β via caspase-8 and pyroptosis via caspase-1 activity.^97^ Overall it appears possible that ASC may recruit caspase-8 to trigger appropriate responses to a microbial insult.

## Death receptors and caspase-8 in autoinflammatory disease

Evidence now suggests that death receptor signalling affects NLRP3 inflammasome activity at three levels; inflammasome priming, activation and assembly, and post-translational modification. First, the RIP kinases and caspase-8 have a critical step in inflammasome priming, as demonstrated by reduced cytokine production and pro-IL-1β in *Ripk3*^−/−^*Caspase-8*^−/−^ mice.^58, 63^ Second, not only can the NLRP3 inflammasome be triggered by RIPK3-caspase-8 and RIPK3-MLKL signalling, but caspase-8 can bind ASC-containing inflammasomes to signal apoptosis and activate IL-1β.^50, 95^ Finally, TNFR1 death receptor signalling molecules have been implicated in NLRP3 inflammasome assembly (that is, c-FLIP_L_),^88^ or post-translational modifications of inflammasome components. For example, LUBAC has been suggested to ubiquitylate the adaptor ASC.^98^ Importantly, mutation of a number of key death receptor components that have previously been linked to autoimmunity or immunodeficiency, also mirror features of autoinflammatory syndromes that are associated with heightened NLRP3 inflammasome activity, IL-1β and/or TNF levels (Table 2).

### TNFR1 and TRAPS

TNF receptor-associated periodic syndrome (TRAPS; Table 2) was one of the first genetically defined autoinflammatory diseases. TRAPS occurs due to autosomal dominant mutations of the TNFR1 *(TNFRS1A)* gene and leads to a hereditary recurrent fever syndrome. Mechanistically, missense mutations in the first two cysteine rich domains of the TNFR1 extracellular domain, critical for receptor association and ligand binding, have been linked to aberrant folding and defective receptor shedding. Defective receptor expression reportedly leads to overexpression, aggregate formation in the endoplasmic reticulum and stress responses, as well as mitochondrial ROS production.^71, 99, 100^ These events have been linked to constitutive TNFR1 signalling and pro-inflammatory cytokine release. Even in a heterozygous state, TRAPS TNFR1-mutant cells exhibit both spontaneous and sustained LPS-induced MAPK activation, resulting in heightened cytokine production, including TNF, IL-6 and IL-1β.^100^ How IL-1β is activated in TRAPS patients remains unclear, yet like TNF inhibition, IL-1 blockade is also used therapeutically.

### XIAP and XLP2

Until recently the E3 ligase XIAP was cast as a direct inhibitor of caspase-3, -7 and -9. However, it has now become abundantly clear in monocyte, macrophages and dendritic cells that XIAP and its E3 ligase activity, has an obligatory role in repressing ripoptosome activation and consequently preventing cell death and IL-1β activation.^18, 50, 78^ Despite functional redundancy of individual IAPs, macrophages lacking XIAP, or XIAP and cIAP2, are sensitised to LPS/TNF-induced RIPK3-caspase-8-mediated apoptosis, RIPK3-MLKL-mediated necroptosis and NLRP3 inflammasome activation.^50^ Of note, myeloid-specific cIAP1 and cIAP2 loss did not sensitise macrophages to death *in vitro* or activate IL-1β, yet mice developed a severe TNF-driven inflammatory arthritis.^50^ In contrast, co-deletion of XIAP with cIAP1 and cIAP2, or inhibition of all three IAPs and TLR/TNFR ligation, resulted in maximal ripoptosome-mediated apoptotic and necroptotic signalling, and IL-1β activation in myeloid cells (Figure 3).^50^ Remarkably, mice lacking all IAPs in myeloid cells exhibited multi-organ inflammation and arthritis associated with heightened inflammatory cytokines, including IL-1β and TNF.^50, 101^

The importance of XIAP in repressing inflammatory signalling in mice is of significant interest, as mutations in *XIAP* are commonly associated with inflammatory bowel disease,^102^ and loss of function mutations in *XIAP* are found in X-linked lymphoproliferative disease 2 (XLP2) patients.^103^ XLP2, which is often associated with Epstein Barr Virus (EBV) infection, results in hemophagocytic lymphohistiocytosis (HLH) in 60–90% of patients.^103^ The characteristic macrophage hyperactivation is associated with elevated levels of the inflammasome-caspase-1 (or caspase-8) substrate IL-18 and clinical features reminiscent of CAPS patients with NLRP3 activating mutations, and mice with myeloid-specific IAP loss (Table 2).^104^ Hence XLP2 with HLH could be reclassified as an inflammasome-driven autoinflammatory syndrome.

### LUBAC and LUBAC autoinflammation

LUBAC, comprised of HOIL-1, HOIP and SHARPIN, is responsible for linear ubiquitylation of RIPK1 and NEMO to allow efficient NF-κB activation. The importance of LUBAC for activating NF-κB signalling in both humans and mice has been demonstrated by blunted cytokine transcription in fibroblasts lacking HOIP, HOIL-1 or SHARPIN in response to TLR ligands, TNF and IL-1β.^9, 105, 106^ In line with this, defects in CD40 ligand signalling have also been observed upon loss of HOIL-1, HOIP and SHARPIN in B cells.^9, 105, 106^ In the myeloid compartment the role of LUBAC in inflammatory signalling is less clear. Similar to the responses seen in fibroblasts, TLR-induced NF-κB activation and cytokine production is defective in dendritic cells and macrophages derived from *Sharpin*^*cpdm/cpdm*^ mice.^105, 107, 108^ However, in contrast, LPS or TNF-induced NF-κB activation and cytokine transcription is normal in HOIL-1-deficient macrophages,^98^ although defective cytokine production has been described upon *Listeria* infection.^109^ Surprisingly, *HOIP* or *HOIL-1* mutations in humans triggers systemic autoinflammation, immunodeficiency and amylopectinosis (Table 2).^105, 106^ Likewise, loss of SHARPIN in mice (*Sharpin*^*cpdm/cpdm*^) causes a TNF/TNFR1 and partly IL-1 driven spontaneous autoinflammatory disease featuring chronic proliferative dermatitis, splenomegaly, and liver inflammation.^9, 110^ In contrast, HOIL-1-deficient mice do not exhibit spontaneous autoinflammation, although animals do develop amylopectin-like deposits in the myocardium with age and exhibit immunodeficiency.^109^ Of note, spontaneous inflammatory responses are triggered in HOIL-1-deficient mice infected with chronic MHV68 and *M. Tuberculosis*.^109^ Consistent with this, *HOIL-1*- and *HOIP*-deficient human monocytes display cytokine hyper-production in response to IL-1β or TNF,^105, 106^ and anti-TNF therapy reduced clinical inflammation in a *HOIL-1* deficient patient.^106^ This contradictory role for LUBAC in autoinflammation highlights the fact that we do not fully appreciate the roles of these proteins on a cell-by-cell basis and in different disease settings.

Epidermal-specific caspase-8 or FADD loss causes necroptotic skin lesions.^45, 111^ Hence, it was somewhat surprising that the dermatitis featured in *Sharpin*^*cpdm/cpdm*^ mice is driven by TNF-induced keratinocyte apoptosis, as shown by the absence of lesions upon epidermal-specific deletion of FADD (on a *Ripk3*^−/−^ background) or heterozygous deletion of caspase-8, but not MLKL loss.^110, 112^ In contrast, other disease manifestations were driven by both apoptotic and necroptotic signalling. For example, MLKL deficiency prevented the leukocytosis, and partly rescued the liver pathology and splenomegaly, in *Sharpin*^*cpdm/cpdm*^ mice.^110^

Considering IL-1 is a pathogenic factor in skin lesion development in SHARPIN-deficient mice, the mechanism behind IL-1β activation was recently evaluated. Deficiency in NLRP3 or caspase-1/11 in *Sharpin*^*cpdm/cpdm*^ mice delayed dermatological symptoms, however, whether NLRP3 inflammasome activity was restricted to keratinocytes versus macrophages remains unclear.^107^ This is particularly puzzlingly, as LUBAC activity appears to be essential for inducing, rather than inhibiting, inflammasome activation in macrophages. HOIL-1, for example, has been implicated in the linear ubiquitiylation of ASC and assembly of ASC-containing AIM2 and NLRP3 inflammasomes.^98^ Although a further study suggested LUBAC component SHARPIN regulated canonical and noncanonical NLRP3 inflammasome priming (that is, pro-IL-1β and caspase-11), and not AIM2 activation.^107^ These discrepancies suggest that inflammasome activation regulation by LUBAC requires further study.

### A20 and HA20

A20 is widely recognised for its ability to dampen TNF/TLR-induced NF-κB signalling via the enzymatic removal of ubiquitin chains from RIPK1, TRAF6 and NEMO. In humans, A20 polymorphisms (reduced A20 protein) are associated with a range of inflammatory diseases, such as rheumatoid arthritis. Recently, inactivating germline mutations in A20 were also discovered to cause a Bechet's like autoinflammatory syndrome, now termed Haploinsufficiency of A20 (HA20). HA20 patients exhibit exaggerated NF-κB responses, constitutive NLRP3 activation and elevated serological inflammatory cytokines, including IL-1β.^113^ Interestingly, deletion of A20 in mice causes early onset lethality due to spontaneous multi-organ inflammation and severe cachexia caused by excessive TLR-MyD88-TRIF-RIPK3 signalling, independent of TNF/TNFR1.^114, 115, 116^ Impressively, cell/tissue-specific A20 deletion also often recapitulates aspects of inflammatory diseases associated with A20 dysfunction. For example, myeloid-specific deletion of A20 causes spontaneous inflammatory arthritis that is dependent on the NLRP3 inflammasome and the IL-1 receptor but not TNF.^117^ These results largely parallel findings in myeloid-specific IAP knockout mice, although IL-1β-associated pathology was TNF-dependent in these animals.^50^ Similar to IAP deficient macrophages, TLR stimulation of A20-deficient macrophages resulted in spontaneous IL-1β activation that was dependent on RIPK3 and partially on NLRP3 inflammasome activation, whereby residual IL-1β was presumably driven by direct cleavage by caspase-8.^50, 117^

How A20 restricts spontaneous TLR-induced RIPK3 mediated NLRP3 activation is unclear. A20 is obviously important in restricting NF-κB transcription and therefore NLRP3 inflammasome priming. Furthermore, A20 reportedly acts downstream to impede NLRP3 signalling by cleaving K63-linked ubiquitin chains from K133 of IL-1β to limit IL-1β processing and secretion.^118^ Additionally, A20 has recently been shown to deubiquitylate RIPK3 on K5 to limit necrosome formation,^116^ however, surprisingly in contrast to RIPK3 loss, MLKL deletion does not protect A20 knockout mice from lethality.^119^ Perhaps RIPK3-caspase-8 inflammatory signalling, rather than necroptotic signalling, drives disease in the absence of A20.

## Death receptor induced caspase-8 signalling and disease

The role of the ripoptosome and caspase-8 signalling in common inflammatory disease and IL-1 activation has been difficult to study due to the early onset embryonic lethality in caspase-8 deficient mice due to excessive necroptotic signalling. Tissue- and cell-restricted deletion of caspase-8 has also been complicated by necroptotic inflammatory phenotypes (Table 1) and the fact caspase-8 regulates cytokine transcription. In fact, loss of key cell death receptor signalling components commonly precipitates in early onset lethality, or increased morbidity due to severe inflammatory disease and tissue destruction (Tables 1 and 2). However, utilising a range of knockout mice and inhibitors of core death machinery the role of the ripoptosome in inflammatory disease is becoming more clear (see Table 3 for recent examples and Khan *et al.*^27^). The discovery that co-deletion of caspase-8 and RIPK3 leads to the birth of viable and healthy animals has led to a greater understanding of the role of caspase-8 signalling has in disease. However, as *Ripk3*^−/−^*Caspase-8*^−/−^ mice eventually develop lymphoproliferative disease (>12 weeks of age), and exhibit defects in TLR-induced cytokine production (ie. pro-IL-1β), results are still difficult to interpret. Nevertheless, caspase-8 cleavage of IL-1β and IL-18, or activation of NLRP3 is likely to be of physiological relevance. For example, the IL-1β dependent murine K/BxN serum transfer arthritis model has been reported to be caspase-1-and MLKL independent, and is instead dependent on RIPK3 and caspase-8 for local and systemic IL-1β secretion during the resolution phase of disease.^50^ Results suggest that caspase-8 activity is required for optimal priming and may directly cleave IL-1β in this model.

The contribution of RIPK3 and necroptotic signalling to a range of diseases reportedly driven by necroptotic signalling was recently re-examined by Newton *et al.*^119^ Contradicting previous findings, no role for RIPK3 and necroptosis was observed in acute pancreatitis, brain injury (major cerebral artery occlusion and hypoxia-induced cerebral oedema) or DSS colitis (Table 3).^119^ Differences in experimental procedures or the microbiome were suggested as plausible explanations for these discrepancies. In contrast, in kidney ischaemia-reperfusion injury and TNF-induced systemic inflammatory syndrome, both MLKL necroptotic and apoptotic caspase-8 signalling contributed to disease pathogenesis.^119^ Of note, the strongest role for necroptosis in disease resides around work exploring the mechanism behind death, rather than NLRP3 activation, induced by viral pathogens (for example, CMV and vaccinia virus) or pore-forming toxin producing bacteria (for example, *Serratia marcescens*).^27, 29, 120^ Collectively these results highlight that previous findings in RIPK3-deficient mice suggesting pathological necroptotic signalling in disease need to be revisited utilising MLKL-deficient mice. Moreover, they suggest that caspase-8 activity is more pivotal in driving inflammatory disease pathologies and IL-1β than previously thought.

## Conclusions

This review summarises an extensive body of research, which suggests there is substantial crosstalk between innate PRR and cell death signalling pathways, and that caspase-8 levels dictate the net outcome. Caspase-8 activity is now recognised to critically suppress necroptosis, regulate cytokine transcription, and to act downstream of RIPK3 or inflammasome-related ASC to induce apoptosis. More remarkably, caspase-8 levels have been shown to act as a rheostat for activation of IL-1β. Ripoptosome-associated caspase-8 can either directly cleave IL-1β or indirectly trigger the NLRP3 inflammasome, and when caspase-8 levels are low RIPK3-MLKL necroptotic signalling can also trigger NLRP3. These findings have major ramifications for our understanding of innate immune cell responses during disease, where apoptotic or necroptotic cell death combined with IL-1β and IL-18 activation could be beneficial for pathogen clearance. Moreover, identification of whether apoptotic versus necroptotic cell death pathways contribute to NLRP3 activation and/or IL-1β activation in common inflammatory disease, and in rare autoinflammatory diseases caused by mutations in cell death regulators, is of major interest and will inform rationale therapeutic drug design.

## Figures and Tables

**Table 1:** Rescue of inflammatory disease phenotypes in mice lacking ripoptosome machinery

*Mouse Model*	*Ripk3*^−/−^	*Ripk1*^−/−^	*Ripk1*^D138N/D138N^	*Mlkl*^−/−^	*Casp8*^−/−^	*FADD*^−/−^	*Tnfr1*^−/−^	*MyD88*^−/−^	*Trif* ^−/−^	*Reference*
Caspase-8 deficiency (*Casp8*^−/−^) Embryonic lethality E10.5	Viable	E19-P0	—	Viable	NA	—	E17	—	No	^28, 32, 33, 43, 119^
Caspase-8 deficiency Intestinal epithelium (*Casp8*^*Villin/ER.cre*^) Ileitis Adult onset	Viable^e^	—	Viable	—	NA	—	—	—	—	^122, 123, 124^
Caspase-8 deficiency epidermis (*Casp8*^*K5/ER.Cre*^) Atopic dermatitis P0–P7	—e	—	—	—	NA	—	P9+^f^	No	No	^45, 123^
Ripk1 deficiency (*Ripk1*^−/−^)	P3-P4^b+^	NA	NA	P3–P4	E19–P0^b^	—	—	P3–P4	—	^41, 42, 43^
Perinatal lethality E19–P0; P2–P5^a^	P2-P21^a,b+,c+^	NA	NA	—	P2–P16^a,b^	—^a,b^	P2–P18^a,c,d+^	—	P2-P7^a,d^	
Ripk1 deficiency intestinal epithelium (*Ripk1*^*Villin.Cre*^) Lethal intestinal disease P1–P30	No^b+^	NA	NA	—	P60+^f,g^	P50+^b,f,g^	P30–P80+^f^	P30-P60+^f^	No^g^	^124, 125^
Ripk1 deficiency epidermis (*Ripk1*^*K14.Cre*^) Atopic dermatitis P8–P21+	Viable^b^	NA	NA	Viable	—	P28+^b+^	P28+	—	P28+	^125^
RIPK3 kinase deficiency (*Ripk3*^*D161N/D161N*^) Embryonic lethality E11.5	NA	E19-P0	No	No	Viable	—	No	—	No	^126^
c-FLIP-deficient (*cflar*^−/−^) Embryonic lethality E10.5	E10.5-E11.5^b+^	—	—	—	—	—b	E14–E17^b^	—	—	^39, 41^
c-FLIP deficiency Intestinal epithelium (*cflar*^*Villin.Cre*^) Fatal intestinal disease P1	No	—	—	—	—	—	P1–P140+ days^f^	—	—	^127^
FADD-deficient (*FADD*^−/−^) Embryonic lethality E10.5	Viable	E19-P0	—	Viable	—	NA	E14–E17	—	—	^39, 40, 128^
FADD deficiency Intestinal Epithelium (*FADD*^*Villin.Cre*^) Intestinal disease P1–P21(50%)	Viable^b^	No^b+,g^	No	—	—	NA	—	—	—	^129^
FADD deficiency epidermis (*FADD*^*K14.Cre*^) Lethal skin lesions P4–P8	Viable^b^	P21+^b+,g^	P21+	—	—	NA	P14–P35	P8–P21	—	^111, 125^
A20 deficiency (*A20*^−/−^) Fatal multi-organ disease P1–P250	P1-P400+	—	P1- 300+	No	—	—	—		—	^119^
cIAP double deficiency (*cIAP1*^−/−^*cIAP2*^−/−^) Embryonic lethality E10	E14.5	E12.5	—	—	—	—	P0–P2	—	—	^130^

Rescue of lethal/inflammation mouse model to E, ∼embryonic day; No, no effect, —, not done; NA, not applicable; P, ∼post-natal day; viable, no lethality or health restored in viable animals.

a *Ripk1*^−/−^129 derived.

b Co-deletion of genes viable.

c,d Enhanced survival with co-deletion.

e Protection shown in adult tissues.

f Delayed or altered disease progression.

g Tissue-specific deletion.

**Table 2:** Autoinflammatory-like syndromes associated with mutations in death receptor signalling machinery

*Death Receptor machinery (aliases)*	*Human disease* *Gene (chromosome position) mutation*	*Mouse model(s)* *Gene mutation*	*Clinical features*	*Reference*
TNFR1 (p55, CD120a)	TNF Receptor-Associated Periodic Fever Syndrome (TRAPS) *TNFRS1A* (12p13.2) High penetrance: C29F/Y, C30Y/R/S/F, C33G/Y, C43R/Y/S, C52W/F/Y/R, C55R/S/Y, C70G/R/S/Y, C88R/Y. Low penetrance: P46L, T50M/K, L57P, S86P and R92Q	*p55*^*ΔNS*^ mice (non-sheddable TNFR1 knock-in) T50M & C33Y TNFR1-mutant mice	Periodic fever Rash Myalgia Arthralgia Abdominal pain Periorbital oedema Lymphadenopathy Serum A protein amyloidosis (kidney)	^71, 100, 131^
XIAP (BIRC4, MIHA, IAP-3)	X-linked Lymphoproliferative disease Type 2 (XLP2) *XIAP* (Xq25) P482R, I494N, G188E, Y290fsX294, Q33X, Q104X, G188E, DelExon6 and DelExon1-5	*Xiap*^−/−^ mice (MHV68-infected) *cIAP1*^*LysM.Cre*^ *Xiap*^−/−^ *cIAP2*^−/−^	Periodic fever Rash Heptosplenomegaly Cytopenia Chronic haemorrhagic colitis Arthralgia	^18, 50, 101, 132^
LUBAC -HOIL-1/HOIP/SHARPIN complex	LUBAC deficiency autoinflammatory disease *HOIL* gene *RBCK1* (20p.13) Biallelic: L41fsX7 Compound heterozygote Q185X/TRIB3:g-1272_HOIL:g9780del, *HOIP* gene *RNF31* (14q12) Biallelic: L72P	*HOIL-1*^−/−^ (MHV68-infected) *Sharpin*^*cpdm/cpdm*^	Periodic fever Rash Hepatosplenomegaly Lymphadenopathy Abdominal pain (IBD) Muscular amylopectinosis (e.g., myocardium, oesophagus, bowel) Lymphangiectasia	^105, 106, 109, 110^
A20 (TNFAIP3)	Haploinsufficiency of A20 (HA20) *A20* gene *TNFAIP3* (6q23.3) High penetrance: F224SfsX4, L227X, P268LfsX19, R271X, Y306X and T604RfsX93	*A20*^−/−^ *A20*^*LysM.Cre*^	Fever Oral ulcers Genital ulcers Rash (erythaema nodosum-like lesions)	^113, 118^

Abbreviations: MHV68, murine gamma herpes virus 68; TNF, tumour necrosis factor.

**Table 3:** Contribution of necroptotic and apoptotic signalling to disease

	*Ripk3*^−/−^	*Ripk1*^*kinase dead*^*/Inhibitor*	*Mlkl*^−/−^*/inhibitor*	*Casp8*^−/−^*/inhibitor*	*Ripk3*^−/−^ *Casp8*^−/−^	*Reference*
*Injury*						
Renal ischaemic reperfusion injury	↓^a^	↓	↓^b^	No effect	↓	d^,119^
Oxalate crystal-induced renal injury^c^	↓	↓	↓	—	—	^133^
Myocardial ischaemic reperfusion injury	↓	↓	—	—	—	d^,119, 133^
Brain Hypoxic Injury	No effect	↓	—	↓	—	d^,119^
Retinal Degeneration^c^	↓	↓	—	No effect	—	d^,134^
Pancreatitis	↓/No effect	↑/No effect	↓	↑	—	d^,119^
						
*Inflammation*						
Imiquimod-induced psoriasis	—	No effect	—	—	—	^119^
K/BxN arthritis^c^	↓^a^	—	No effect	—	↓	^50^
DSS colitis^c^	↑/No effect	↓/No effect	—	—	—	^59, 119^
EAE	—	↓	—	—	—	^52^
OVA-induced asthma^c^	No effect	—	—	—	↓	^135^
Cecal ligation and puncture	No effect	↓	No effect	↓	—	d
TNF-induced	↓	↓/↑	↓	↓/↑	↓	d^,119^
LPS-induced	↓/No effect	No effect	No effect	↑	↓	d^,58, 119^
						
*Infectious disease*						
Murine cytomegalovirus	↑	—	—	↑	—	d^,29^
Vaccinia virus	↑	—	—	—	—	^d,29^
HSV-1	↑	—	—	—	—	^136^
Influenza A virus	↑	—	No effect	—	—	^137^
*Serratia marcescens*^c^	↓	↓	↓	—	—	^120^
*Staphylococcus aureus*^c^	↓	↓	—	—	—	^138^
*Yersinia pestis*^c^	No effect	—	—	—	↑	^63, 90^

Abbreviations: DSS dextran sulphate sodium; EAE experimental autoimmune encephalomyelitis; HSV-1 herpes simplex virus-1; OVA ovalbumin.

Key, Not done,↓, reduced disease,↑, increased disease.

a *Ripk3*^−/−^ *Caspase-8*^−/−^ show equivalent protection to Ripk3^−/−^.

b Partial contribution.

c NLRP3 inflammasome or caspase-8 activity implicated in IL-1β activation in study.

d Original references in Khan *et al.*^27^ due to space constraints.

## References

[bib1] Speir M, Lawlor KE, Glaser SP, Abraham G, Chow S, Vogrin A et al. Eliminating Legionella by inhibiting BCL-XL to induce macrophage apoptosis. Nat Microbiol 2016; 1: 15034. 27572165 10.1038/nmicrobiol.2015.34

[bib2] Ebert G, Allison C, Preston S, Cooney J, Toe JG, Stutz MD et al. Eliminating hepatitis B by antagonizing cellular inhibitors of apoptosis. Proc Natl Acad Sci USA 2015; 112: 5803–5808. 25902530 10.1073/pnas.1502400112 PMC4426438

[bib3] Su HC, Lenardo MJ. Genetic defects of apoptosis and primary immunodeficiency. Immunol Allergy Clin North Am 2008; 28: 329–351. 18424336 10.1016/j.iac.2008.01.002 PMC2671802

[bib4] Delbridge AR, Grabow S, Strasser A, Vaux DL. Thirty years of BCL-2: translating cell death discoveries into novel cancer therapies. Nat Rev Cancer 2016; 16: 99–109. 26822577 10.1038/nrc.2015.17

[bib5] Sedger LM, McDermott MF. TNF and TNF-receptors: From mediators of cell death and inflammation to therapeutic giants—past, present and future. Cytokine Growth Factor Rev 2014; 25: 453–472. 25169849 10.1016/j.cytogfr.2014.07.016

[bib6] Mitoma H, Horiuchi T, Tsukamoto H, Tamimoto Y, Kimoto Y, Uchino A et al. Mechanisms for cytotoxic effects of anti-tumor necrosis factor agents on transmembrane tumor necrosis factor alpha-expressing cells: comparison among infliximab, etanercept, and adalimumab. Arthritis Rheum 2008; 58: 1248–1257. 18438840 10.1002/art.23447

[bib7] Micheau O, Tschopp J. Induction of TNF receptor I-mediated apoptosis via two sequential signaling complexes. Cell 2003; 114: 181–190. 12887920 10.1016/s0092-8674(03)00521-x

[bib8] Haas TL, Emmerich CH, Gerlach B, Schmukle AC, Cordier SM, Rieser E et al. Recruitment of the linear ubiquitin chain assembly complex stabilizes the TNF-R1 signaling complex and is required for TNF-mediated gene induction. Mol Cell 2009; 36: 831–844. 20005846 10.1016/j.molcel.2009.10.013

[bib9] Gerlach B, Cordier SM, Schmukle AC, Emmerich CH, Rieser E, Haas TL et al. Linear ubiquitination prevents inflammation and regulates immune signalling. Nature 2011; 471: 591–596. 21455173 10.1038/nature09816

[bib10] Hsu H, Xiong J, Goeddel DV. The TNF receptor 1-associated protein TRADD signals cell death and NF-kappa B activation. Cell 1995; 81: 495–504. 7758105 10.1016/0092-8674(95)90070-5

[bib11] Vince JE, Pantaki D, Feltham R, Mace PD, Cordier SM, Schmukle AC et al. TRAF2 must bind to cellular inhibitors of apoptosis for tumor necrosis factor (tnf) to efficiently activate nf-{kappa}b and to prevent tnf-induced apoptosis. J Biol Chem 2009; 284: 35906–35915. 19815541 10.1074/jbc.M109.072256 PMC2791019

[bib12] Iwai K, Fujita H, Sasaki Y. Linear ubiquitin chains: NF-kappaB signalling, cell death and beyond. Nat Rev Mol Cell Biol 2014; 15: 503–508. 25027653 10.1038/nrm3836

[bib13] Tsuchiya Y, Nakabayashi O, Nakano H. FLIP the switch: regulation of apoptosis and necroptosis by cFLIP. Int J Mol Sci 2015; 16: 30321–30341. 26694384 10.3390/ijms161226232 PMC4691174

[bib14] Hildebrand JM, Tanzer MC, Lucet IS, Young SN, Spall SK, Sharma P et al. Activation of the pseudokinase MLKL unleashes the four-helix bundle domain to induce membrane localization and necroptotic cell death. Proc Natl Acad Sci USA 2014; 111: 15072–15077. 25288762 10.1073/pnas.1408987111 PMC4210347

[bib15] Murphy JM, Czabotar PE, Hildebrand JM, Lucet IS, Zhang JG, Alvarez-Diaz S et al. The pseudokinase MLKL mediates necroptosis via a molecular switch mechanism. Immunity 2013; 39: 443–453. 24012422 10.1016/j.immuni.2013.06.018

[bib16] Sun L, Wang H, Wang Z, He S, Chen S, Liao D et al. Mixed lineage kinase domain-like protein mediates necrosis signaling downstream of RIP3 kinase. Cell 2012; 148: 213–227. 22265413 10.1016/j.cell.2011.11.031

[bib17] Dondelinger Y, Declercq W, Montessuit S, Roelandt R, Goncalves A, Bruggeman I et al. MLKL compromises plasma membrane integrity by binding to phosphatidylinositol phosphates. Cell Rep 2014; 7: 971–981. 24813885 10.1016/j.celrep.2014.04.026

[bib18] Yabal M, Muller N, Adler H, Knies N, Gross CJ, Damgaard RB et al. XIAP restricts TNF- and RIP3-dependent cell death and inflammasome activation. Cell Rep 2014; 7: 1796–1808. 24882010 10.1016/j.celrep.2014.05.008

[bib19] Tenev T, Bianchi K, Darding M, Broemer M, Langlais C, Wallberg F et al. The ripoptosome, a signaling platform that assembles in response to genotoxic stress and loss of IAPs. Mol Cell 2011; 43: 432–448. 21737329 10.1016/j.molcel.2011.06.006

[bib20] Feoktistova M, Geserick P, Kellert B, Dimitrova DP, Langlais C, Hupe M et al. cIAPs block ripoptosome formation, a RIP1/caspase-8 containing intracellular cell death complex differentially regulated by cFLIP isoforms. Mol Cell 2011; 43: 449–463. 21737330 10.1016/j.molcel.2011.06.011 PMC3163271

[bib21] Cullen SP, Henry CM, Kearney CJ, Logue SE, Feoktistova M, Tynan GA et al. Fas/CD95-induced chemokines can serve as ‘find-me' signals for apoptotic cells. Mol Cell 2013; 49: 1034–1048. 23434371 10.1016/j.molcel.2013.01.025

[bib22] Berg D, Stuhmer T, Siegmund D, Muller N, Giner T, Dittrich-Breiholz O et al. Oligomerized tumor necrosis factor-related apoptosis inducing ligand strongly induces cell death in myeloma cells, but also activates proinflammatory signaling pathways. FEBS J 2009; 276: 6912–6927. 19895579 10.1111/j.1742-4658.2009.07388.x

[bib23] Park DR, Thomsen AR, Frevert CW, Pham U, Skerrett SJ, Kiener PA et al. Fas (CD95) induces proinflammatory cytokine responses by human monocytes and monocyte-derived macrophages. J Immunol 2003; 170: 6209–6216. 12794152 10.4049/jimmunol.170.12.6209

[bib24] Kallenberger SM, Beaudouin J, Claus J, Fischer C, Sorger PK, Legewie S et al. Intra- and interdimeric caspase-8 self-cleavage controls strength and timing of CD95-induced apoptosis. Sci Signal 2014; 7: ra23. 24619646 10.1126/scisignal.2004738 PMC4208692

[bib25] Pop C, Oberst A, Drag M, Van Raam BJ, Riedl SJ, Green DR et al. FLIP(L) induces caspase 8 activity in the absence of interdomain caspase 8 cleavage and alters substrate specificity. Biochem J 2011; 433: 447–457. 21235526 10.1042/BJ20101738 PMC4024219

[bib26] Walczak H. Death receptor-ligand systems in cancer, cell death and inflammation. Cold Spring Harb Perspect Biol 2013; 5: a008698. 23637280 10.1101/cshperspect.a008698 PMC3632055

[bib27] Khan N, Lawlor KE, Murphy JM, Vince JE. More to life than death: molecular determinants of necroptotic and non-necroptotic RIP3 kinase signaling. Curr Opin Immunol 2014; 26: 76–89. 24556404 10.1016/j.coi.2013.10.017

[bib28] Kaiser WJ, Sridharan H, Huang C, Mandal P, Upton JW, Gough PJ et al. Toll-like receptor 3-mediated necrosis via TRIF, RIP3 and MLKL. J Biol Chem 2013; 288: 31268–31279. 24019532 10.1074/jbc.M113.462341 PMC3829437

[bib29] Mocarski ES, Guo H, Kaiser WJ. Necroptosis: The Trojan horse in cell autonomous antiviral host defense. Virology 2015; 479–480: 160–166. 10.1016/j.virol.2015.03.016 PMC5115625 25819165

[bib30] He S, Liang Y, Shao F, Wang X. Toll-like receptors activate programmed necrosis in macrophages through a receptor-interacting kinase-3-mediated pathway. Proc Natl Acad Sci USA 2011; 108: 20054–20059. 22123964 10.1073/pnas.1116302108 PMC3250173

[bib31] Varfolomeev EE, Schuchmann M, Luria V, Chiannilkulchai N, Beckmann JS, Mett IL et al. Targeted disruption of the mouse Caspase 8 gene ablates cell death induction by the TNF receptors, Fas/Apo1, and DR3 and is lethal prenatally. Immunity 1998; 9: 267–276. 9729047 10.1016/s1074-7613(00)80609-3

[bib32] Oberst A, Dillon CP, Weinlich R, McCormick LL, Fitzgerald P, Pop C et al. Catalytic activity of the caspase-8-FLIP(L) complex inhibits RIPK3-dependent necrosis. Nature 2011; 471: 363–367. 21368763 10.1038/nature09852 PMC3077893

[bib33] Kaiser WJ, Upton JW, Long AB, Livingston-Rosanoff D, Daley-Bauer LP, Hakem R et al. RIP3 mediates the embryonic lethality of caspase-8-deficient mice. Nature 2011; 471: 368–372. 21368762 10.1038/nature09857 PMC3060292

[bib34] Alvarez-Diaz S, Dillon CP, Lalaoui N, Tanzer MC, Rodriguez DA, Lin A et al. The pseudokinase MLKL and the kinase RIPK3 have distinct roles in autoimmune disease caused by loss of death-receptor-induced apoptosis. Immunity 2016; 45: 513–526. 27523270 10.1016/j.immuni.2016.07.016 PMC5040700

[bib35] Watanabe-Fukunaga R, Brannan CI, Copeland NG, Jenkins NA, Nagata S. Lymphoproliferation disorder in mice explained by defects in Fas antigen that mediates apoptosis. Nature 1992; 356: 314–317. 1372394 10.1038/356314a0

[bib36] Yeh WC, Pompa JL, McCurrach ME, Shu HB, Elia AJ, Shahinian A et al. FADD: essential for embryo development and signaling from some, but not all, inducers of apoptosis. Science 1998; 279: 1954–1958. 9506948 10.1126/science.279.5358.1954

[bib37] Zhang J, Cado D, Chen A, Kabra NH, Winoto A. Fas-mediated apoptosis and activation-induced T-cell proliferation are defective in mice lacking FADD/Mort1. Nature 1998; 392: 296–300. 9521326 10.1038/32681

[bib38] Yeh WC, Itie A, Elia AJ, Ng M, Shu HB, Wakeham A et al. Requirement for Casper (c-FLIP) in regulation of death receptor-induced apoptosis and embryonic development. Immunity 2000; 12: 633–642. 10894163 10.1016/s1074-7613(00)80214-9

[bib39] Dillon CP, Oberst A, Weinlich R, Janke LJ, Kang TB, Ben-Moshe T et al. Survival function of the FADD-CASPASE-8-cFLIP(L) complex. Cell Rep 2012; 1: 401–407. 22675671 10.1016/j.celrep.2012.03.010 PMC3366463

[bib40] Zhang X, Fan C, Zhang H, Zhao Q, Liu Y, Xu C et al. MLKL and FADD are critical for suppressing progressive lymphoproliferative disease and activating the NLRP3 inflammasome. Cell Rep 2016; 16: 3247–3259. 27498868 10.1016/j.celrep.2016.06.103 PMC7191534

[bib41] Dillon CP, Weinlich R, Rodriguez DA, Cripps JG, Quarato G, Gurung P et al. RIPK1 blocks early postnatal lethality mediated by caspase-8 and RIPK3. Cell 2014; 157: 1189–1202. 24813850 10.1016/j.cell.2014.04.018 PMC4068710

[bib42] Kaiser WJ, Daley-Bauer LP, Thapa RJ, Mandal P, Berger SB, Huang C et al. RIP1 suppresses innate immune necrotic as well as apoptotic cell death during mammalian parturition. Proc Natl Acad Sci USA 2014; 111: 7753–7758. 24821786 10.1073/pnas.1401857111 PMC4040608

[bib43] Rickard JA, O'Donnell JA, Evans JM, Lalaoui N, Poh AR, Rogers TW et al. RIPK1 regulates RIPK3-MLKL driven systemic inflammation and emergency hematopoiesis. Cell 2014; 157: 1175–1188. 24813849 10.1016/j.cell.2014.04.019

[bib44] Günther C, Buchen B, He G-W, Hornef M, Torow N, Neumann H et al. Caspase-8 controls the gut response to microbial challenges by Tnf-α-dependent and independent pathways. Gut 2015; 64: 601–610. 25379949 10.1136/gutjnl-2014-307226 PMC4392221

[bib45] Kovalenko A, Kim JC, Kang TB, Rajput A, Bogdanov K, Dittrich-Breiholz O et al. Caspase-8 deficiency in epidermal keratinocytes triggers an inflammatory skin disease. J Exp Med 2009; 206: 2161–2177. 19720838 10.1084/jem.20090616 PMC2757876

[bib46] O'Reilly LA, Tai L, Lee L, Kruse EA, Grabow S, Fairlie WD et al. Membrane-bound Fas ligand only is essential for Fas-induced apoptosis. Nature 2009; 461: 659–663. 19794494 10.1038/nature08402 PMC2785124

[bib47] Low JT, Hughes P, Lin A, Siebenlist U, Jain R, Yaprianto K et al. Impact of loss of NF-kappaB1, NF-kappaB2 or c-REL on SLE-like autoimmune disease and lymphadenopathy in Fas(lpr/lpr) mutant mice. Immunol Cell Biol 2016; 94: 66–78. 26084385 10.1038/icb.2015.66

[bib48] Ch'en IL, Tsau JS, Molkentin JD, Komatsu M, Hedrick SM. Mechanisms of necroptosis in T cells. J Exp Med 2011; 208: 633–641. 21402742 10.1084/jem.20110251 PMC3135356

[bib49] Kang TB, Yang SH, Toth B, Kovalenko A, Wallach D. Caspase-8 Blocks Kinase RIPK3-Mediated Activation of the NLRP3 Inflammasome. Immunity 2013; 38: 27–40. 23260196 10.1016/j.immuni.2012.09.015

[bib50] Lawlor KE, Khan N, Mildenhall A, Gerlic M, Croker BA, D'Cruz AA et al. RIPK3 promotes cell death and NLRP3 inflammasome activation in the absence of MLKL. Nat Commun 2015; 6: 6282. 25693118 10.1038/ncomms7282 PMC4346630

[bib51] Li J, McQuade T, Siemer AB, Napetschnig J, Moriwaki K, Hsiao YS et al. The RIP1/RIP3 necrosome forms a functional amyloid signaling complex required for programmed necrosis. Cell 2012; 150: 339–350. 22817896 10.1016/j.cell.2012.06.019 PMC3664196

[bib52] Ofengeim D, Ito Y, Najafov A, Zhang Y, Shan B, DeWitt JP et al. Activation of necroptosis in multiple sclerosis. Cell Rep 2015; 10: 1836–1849. 25801023 10.1016/j.celrep.2015.02.051 PMC4494996

[bib53] Hughes MA, Powley IR, Jukes-Jones R, Horn S, Feoktistova M, Fairall L et al. Co-operative and hierarchical binding of c-FLIP and caspase-8: a unified model defines how c-FLIP isoforms differentially control cell fate. Mol Cell 2016; 61: 834–849. 26990987 10.1016/j.molcel.2016.02.023 PMC4819448

[bib54] O'Donnell MA, Perez-Jimenez E, Oberst A, Ng A, Massoumi R, Xavier R et al. Caspase 8 inhibits programmed necrosis by processing CYLD. Nat Cell Biol 2011; 13: 1437–1442. 22037414 10.1038/ncb2362 PMC3229661

[bib55] Legarda D, Justus SJ, Ang RL, Rikhi N, Li W, Moran TM et al. CYLD proteolysis protects macrophages from TNF-mediated auto-necroptosis induced by LPS and licensed by type I IFN. Cell Rep 2016; 15: 2449–2461. 27264187 10.1016/j.celrep.2016.05.032 PMC4909532

[bib56] McComb S, Cessford E, Alturki NA, Joseph J, Shutinoski B, Startek JB et al. Type-I interferon signaling through ISGF3 complex is required for sustained Rip3 activation and necroptosis in macrophages. Proc Natl Acad Sci USA 2014; 111: E3206–E3213. 25049377 10.1073/pnas.1407068111 PMC4128105

[bib57] Wong WW, Gentle IE, Nachbur U, Anderton H, Vaux DL, Silke J. RIPK1 is not essential for TNFR1-induced activation of NF-kappaB. Cell Death Differ 2010; 17: 482–487. 19927158 10.1038/cdd.2009.178

[bib58] Allam R, Lawlor KE, Yu EC, Mildenhall AL, Moujalled DM, Lewis RS et al. Mitochondrial apoptosis is dispensable for NLRP3 inflammasome activation but non-apoptotic caspase-8 is required for inflammasome priming. EMBO Rep 2014; 15: 982–990. 24990442 10.15252/embr.201438463 PMC4198042

[bib59] Moriwaki K, Balaji S, McQuade T, Malhotra N, Kang J, Chan FK. The necroptosis adaptor RIPK3 promotes injury-induced cytokine expression and tissue repair. Immunity 2014; 41: 567–578. 25367573 10.1016/j.immuni.2014.09.016 PMC4220270

[bib60] Cuda CM, Misharin AV, Gierut AK, Saber R, Haines GK 3rd, Hutcheson J et al. Caspase-8 acts as a molecular rheostat to limit RIPK1- and MyD88-mediated dendritic cell activation. J Immunol 2014; 192: 5548–5560. 24808358 10.4049/jimmunol.1400122 PMC4074511

[bib61] Cuda CM, Misharin AV, Khare S, Saber R, Tsai F, Archer AM et al. Conditional deletion of caspase-8 in macrophages alters macrophage activation in a RIPK-dependent manner. Arthritis Res Ther 2015; 17: 291. 26471282 10.1186/s13075-015-0794-z PMC4608154

[bib62] Gurung P, Anand PK, Malireddi RK, Vande Walle L, Van Opdenbosch N, Dillon CP et al. FADD and caspase-8 mediate priming and activation of the canonical and noncanonical Nlrp3 inflammasomes. J Immunol 2014; 192: 1835–1846. 24453255 10.4049/jimmunol.1302839 PMC3933570

[bib63] Weng D, Marty-Roix R, Ganesan S, Proulx MK, Vladimer GI, Kaiser WJ et al. Caspase-8 and RIP kinases regulate bacteria-induced innate immune responses and cell death. Proc Natl Acad Sci USA 2014; 111: 7391–7396. 24799678 10.1073/pnas.1403477111 PMC4034196

[bib64] Broz P, Dixit VM. Inflammasomes: mechanism of assembly, regulation and signalling. Nat Rev Immunol 2016; 16: 407–420. 27291964 10.1038/nri.2016.58

[bib65] Kayagaki N, Warming S, Lamkanfi M, Vande Walle L, Louie S, Dong J et al. Non-canonical inflammasome activation targets caspase-11. Nature 2011; 479: 117–121. 22002608 10.1038/nature10558

[bib66] Shi J, Zhao Y, Wang Y, Gao W, Ding J, Li P et al. Inflammatory caspases are innate immune receptors for intracellular LPS. Nature 2014; 514: 187–192. 25119034 10.1038/nature13683

[bib67] Shi J, Zhao Y, Wang K, Shi X, Wang Y, Huang H et al. Cleavage of GSDMD by inflammatory caspases determines pyroptotic cell death. Nature 2015; 526: 660–665. 26375003 10.1038/nature15514

[bib68] Yang D, He Y, Munoz-Planillo R, Liu Q, Nunez G. Caspase-11 requires the pannexin-1 channel and the purinergic P2X7 pore to mediate pyroptosis and endotoxic shock. Immunity 2015; 43: 923–932. 26572062 10.1016/j.immuni.2015.10.009 PMC4795157

[bib69] Xu H, Yang J, Gao W, Li L, Li P, Zhang L et al. Innate immune sensing of bacterial modifications of Rho GTPases by the Pyrin inflammasome. Nature 2014; 513: 237–241. 24919149 10.1038/nature13449

[bib70] Menu P, Vince JE. The NLRP3 inflammasome in health and disease: the good, the bad and the ugly. Clin Exp Immunol 2011; 166: 1–15. 21762124 10.1111/j.1365-2249.2011.04440.x PMC3193914

[bib71] Aksentijevich I, Kastner DL. Genetics of monogenic autoinflammatory diseases: past successes, future challenges. Nat Rev Rheumatol 2011; 7: 469–478. 21727933 10.1038/nrrheum.2011.94

[bib72] Munoz-Planillo R, Kuffa P, Martinez-Colon G, Smith BL, Rajendiran TM, Nunez G. K(+) efflux is the common trigger of NLRP3 inflammasome activation by bacterial toxins and particulate matter. Immunity 2013; 38: 1142–1153. 23809161 10.1016/j.immuni.2013.05.016 PMC3730833

[bib73] Wolf AJ, Reyes CN, Liang W, Becker C, Shimada K, Wheeler ML et al. Hexokinase is an innate immune receptor for the detection of bacterial peptidoglycan. Cell 2016; 166: 624–636. 27374331 10.1016/j.cell.2016.05.076 PMC5534359

[bib74] Gaidt MM, Ebert TS, Chauhan D, Schmidt T, Schmid-Burgk JL, Rapino F et al. Human monocytes engage an alternative inflammasome pathway. Immunity 2016; 44: 833–846. 27037191 10.1016/j.immuni.2016.01.012

[bib75] Lawlor KE, Vince JE. Ambiguities in NLRP3 inflammasome regulation: is there a role for mitochondria? Biochim Biophys Acta 2014; 1840: 1433–1440. 23994495 10.1016/j.bbagen.2013.08.014

[bib76] Iyer SS, He Q, Janczy JR, Elliott EI, Zhong Z, Olivier AK et al. Mitochondrial cardiolipin is required for Nlrp3 inflammasome activation. Immunity 2013; 39: 311–323. 23954133 10.1016/j.immuni.2013.08.001 PMC3779285

[bib77] Maelfait J, Vercammen E, Janssens S, Schotte P, Haegman M, Magez S et al. Stimulation of Toll-like receptor 3 and 4 induces interleukin-1beta maturation by caspase-8. J Exp Med 2008; 205: 1967–1973. 18725521 10.1084/jem.20071632 PMC2526192

[bib78] Vince JE, Wong WW, Gentle I, Lawlor KE, Allam R, O'Reilly L et al. Inhibitor of apoptosis proteins limit RIP3 kinase-dependent interleukin-1 activation. Immunity 2012; 36: 215–227. 22365665 10.1016/j.immuni.2012.01.012

[bib79] Moriwaki K, Bertin J, Gough PJ, Chan FK. A RIPK3-caspase 8 complex mediates atypical pro-IL-1beta processing. J Immunol 2015; 194: 1938–1944. 25567679 10.4049/jimmunol.1402167 PMC4324020

[bib80] Bossaller L, Chiang PI, Schmidt-Lauber C, Ganesan S, Kaiser WJ, Rathinam VA et al. Cutting edge: FAS (CD95) mediates noncanonical IL-1beta and IL-18 maturation via caspase-8 in an RIP3-independent manner. J Immunol 2012; 189: 5508–5512. 23144495 10.4049/jimmunol.1202121 PMC3518757

[bib81] Hedl M, Abraham C. A TNFSF15 disease-risk polymorphism increases pattern-recognition receptor-induced signaling through caspase-8-induced IL-1. Proc Natl Acad Sci USA 2014; 111: 13451–13456. 25197060 10.1073/pnas.1404178111 PMC4169936

[bib82] Gringhuis SI, Kaptein TM, Wevers BA, Theelen B, van der Vlist M, Boekhout T et al. Dectin-1 is an extracellular pathogen sensor for the induction and processing of IL-1beta via a noncanonical caspase-8 inflammasome. Nat Immunol 2012; 13: 246–254. 22267217 10.1038/ni.2222

[bib83] Shenderov K, Riteau N, Yip R, Mayer-Barber KD, Oland S, Hieny S et al. Cutting edge: Endoplasmic reticulum stress licenses macrophages to produce mature IL-1beta in response to TLR4 stimulation through a caspase-8- and TRIF-dependent pathway. J Immunol 2014; 192: 2029–2033. 24489101 10.4049/jimmunol.1302549 PMC3935725

[bib84] Antonopoulos C, El Sanadi C, Kaiser WJ, Mocarski ES, Dubyak GR. Proapoptotic chemotherapeutic drugs induce noncanonical processing and release of IL-1beta via caspase-8 in dendritic cells. J Immunol 2013; 191: 4789–4803. 24078693 10.4049/jimmunol.1300645 PMC3870469

[bib85] Stammler D, Eigenbrod T, Menz S, Frick JS, Sweet MJ, Shakespear MR et al. Inhibition of histone deacetylases permits lipopolysaccharide-mediated secretion of bioactive IL-1beta via a caspase-1-independent mechanism. J Immunol 2015; 195: 5421–5431. 26519528 10.4049/jimmunol.1501195

[bib86] Gross O, Poeck H, Bscheider M, Dostert C, Hannesschläger N, Endres S et al. Syk kinase signalling couples to the Nlrp3 inflammasome for anti-fungal host defence. Nature 2009; 459: 433–436. 19339971 10.1038/nature07965

[bib87] Ganesan S, Rathinam VA, Bossaller L, Army K, Kaiser WJ, Mocarski ES et al. Caspase-8 modulates dectin-1 and complement receptor 3-driven IL-1beta production in response to beta-glucans and the fungal pathogen, Candida albicans. J Immunol 2014; 193: 2519–2530. 25063877 10.4049/jimmunol.1400276 PMC4134963

[bib88] Wu YH, Kuo WC, Wu YJ, Yang KT, Chen ST, Jiang ST et al. Participation of c-FLIP in NLRP3 and AIM2 inflammasome activation. Cell Death Differ 2014; 21: 451–461. 24270411 10.1038/cdd.2013.165 PMC3921593

[bib89] Antonopoulos C, Russo HM, El Sanadi C, Martin BN, Li X, Kaiser WJ et al. Caspase-8 as an effector and regulator of NLRP3 inflammasome signaling. J Biol Chem 2015; 290: 20167–20184. 26100631 10.1074/jbc.M115.652321 PMC4536427

[bib90] Philip NH, Dillon CP, Snyder AG, Fitzgerald P, Wynosky-Dolfi MA, Zwack EE et al. Caspase-8 mediates caspase-1 processing and innate immune defense in response to bacterial blockade of NF-kappaB and MAPK signaling. Proc Natl Acad Sci USA 2014; 111: 7385–7390. 24799700 10.1073/pnas.1403252111 PMC4034241

[bib91] Mandal P, Berger SB, Pillay S, Moriwaki K, Huang C, Guo H et al. RIP3 induces apoptosis independent of pronecrotic kinase activity. Mol Cell 2014; 56: 481–495. 25459880 10.1016/j.molcel.2014.10.021 PMC4512186

[bib92] Kang S, Fernandes-Alnemri T, Rogers C, Mayes L, Wang Y, Dillon C et al. Caspase-8 scaffolding function and MLKL regulate NLRP3 inflammasome activation downstream of TLR3. Nat Commun 2015; 6: 7515. 26104484 10.1038/ncomms8515 PMC4480782

[bib93] Vajjhala PR, Lu A, Brown DL, Pang SW, Sagulenko V, Sester DP et al. The inflammasome adaptor ASC induces procaspase-8 death effector domain filaments. J Biol Chem 2015; 290: 29217–29230. 26468282 10.1074/jbc.M115.687731 PMC4705927

[bib94] Sagulenko V, Thygesen SJ, Sester DP, Idris A, Cridland JA, Vajjhala PR et al. AIM2 and NLRP3 inflammasomes activate both apoptotic and pyroptotic death pathways via ASC. Cell Death Differ 2013; 20: 1149–1160. 23645208 10.1038/cdd.2013.37 PMC3741496

[bib95] Pierini R, Juruj C, Perret M, Jones CL, Mangeot P, Weiss DS et al. AIM2/ASC triggers caspase-8-dependent apoptosis in Francisella-infected caspase-1-deficient macrophages. Cell Death Differ 2012; 19: 1709–1721. 22555457 10.1038/cdd.2012.51 PMC3438500

[bib96] Pierini R, Perret M, Djebali S, Juruj C, Michallet MC, Forster I et al. ASC controls IFN-gamma levels in an IL-18-dependent manner in caspase-1-deficient mice infected with Francisella novicida. J Immunol 2013; 191: 3847–3857. 23975862 10.4049/jimmunol.1203326

[bib97] Man SM, Tourlomousis P, Hopkins L, Monie TP, Fitzgerald KA, Bryant CE. Salmonella infection induces recruitment of Caspase-8 to the inflammasome to modulate IL-1beta production. J Immunol 2013; 191: 5239–5246. 24123685 10.4049/jimmunol.1301581 PMC3835177

[bib98] Rodgers MA, Bowman JW, Fujita H, Orazio N, Shi M, Liang Q et al. The linear ubiquitin assembly complex (LUBAC) is essential for NLRP3 inflammasome activation. J Exp Med 2014; 211: 1333–1347. 24958845 10.1084/jem.20132486 PMC4076580

[bib99] Bulua AC, Simon A, Maddipati R, Pelletier M, Park H, Kim KY et al. Mitochondrial reactive oxygen species promote production of proinflammatory cytokines and are elevated in TNFR1-associated periodic syndrome (TRAPS). J Exp Med 2011; 208: 519–533. 21282379 10.1084/jem.20102049 PMC3058571

[bib100] Simon A, Park H, Maddipati R, Lobito AA, Bulua AC, Jackson AJ et al. Concerted action of wild-type and mutant TNF receptors enhances inflammation in TNF receptor 1-associated periodic fever syndrome. Proc Natl Acad Sci USA 2010; 107: 9801–9806. 20457915 10.1073/pnas.0914118107 PMC2906866

[bib101] Wong WW, Vince JE, Lalaoui N, Lawlor KE, Chau D, Bankovacki A et al. cIAPs and XIAP regulate myelopoiesis through cytokine production in an RIPK1- and RIPK3-dependent manner. Blood 2014; 123: 2562–2572. 24497535 10.1182/blood-2013-06-510743

[bib102] Zeissig Y, Petersen BS, Milutinovic S, Bosse E, Mayr G, Peuker K et al. XIAP variants in male Crohn's disease. Gut 2015; 64: 66–76. 24572142 10.1136/gutjnl-2013-306520

[bib103] Yang X, Miyawaki T, Kanegane H. SAP and XIAP deficiency in hemophagocytic lymphohistiocytosis. Pediatr Int 2012; 54: 447–454. 22672194 10.1111/j.1442-200X.2012.03683.x

[bib104] Wada T, Kanegane H, Ohta K, Katoh F, Imamura T, Nakazawa Y et al. Sustained elevation of serum interleukin-18 and its association with hemophagocytic lymphohistiocytosis in XIAP deficiency. Cytokine 2014; 65: 74–78. 24084330 10.1016/j.cyto.2013.09.007

[bib105] Boisson B, Laplantine E, Dobbs K, Cobat A, Tarantino N, Hazen M et al. Human HOIP and LUBAC deficiency underlies autoinflammation, immunodeficiency, amylopectinosis, and lymphangiectasia. J Exp Med 2015; 212: 939–951. 26008899 10.1084/jem.20141130 PMC4451137

[bib106] Boisson B, Laplantine E, Prando C, Giliani S, Israelsson E, Xu Z et al. Immunodeficiency, autoinflammation and amylopectinosis in humans with inherited HOIL-1 and LUBAC deficiency. Nat Immunol 2012; 13: 1178–1186. 23104095 10.1038/ni.2457 PMC3514453

[bib107] Gurung P, Lamkanfi M, Kanneganti TD. Cutting edge: SHARPIN is required for optimal NLRP3 inflammasome activation. J Immunol 2015; 194: 2064–2067. 25637014 10.4049/jimmunol.1402951 PMC4340749

[bib108] Wang Z, Potter CS, Sundberg JP, Hogenesch H. SHARPIN is a key regulator of immune and inflammatory responses. J Cell Mol Med 2012; 16: 2271–2279. 22452937 10.1111/j.1582-4934.2012.01574.x PMC3402681

[bib109] MacDuff DA, Reese TA, Kimmey JM, Weiss LA, Song C, Zhang X et al. Phenotypic complementation of genetic immunodeficiency by chronic herpesvirus infection. Elife 2015; 4: e04494. 10.7554/eLife.04494 PMC4298697 25599590

[bib110] Rickard JA, Anderton H, Etemadi N, Nachbur U, Darding M, Peltzer N et al. TNFR1-dependent cell death drives inflammation in Sharpin-deficient mice. Elife 2014; 3: e03464. 10.7554/eLife.03464 PMC4270099 25443632

[bib111] Bonnet MC, Preukschat D, Welz PS, van Loo G, Ermolaeva MA, Bloch W et al. The adaptor protein FADD protects epidermal keratinocytes from necroptosis *in vivo* and prevents skin inflammation. Immunity 2011; 35: 572–582. 22000287 10.1016/j.immuni.2011.08.014

[bib112] Kumari S, Redouane Y, Lopez-Mosqueda J, Shiraishi R, Romanowska M, Lutzmayer S et al. Sharpin prevents skin inflammation by inhibiting TNFR1-induced keratinocyte apoptosis. Elife 2014; 3: e03422. 10.7554/eLife.03422 PMC4225491 25443631

[bib113] Zhou Q, Wang H, Schwartz DM, Stoffels M, Park YH, Zhang Y et al. Loss-of-function mutations in TNFAIP3 leading to A20 haploinsufficiency cause an early-onset autoinflammatory disease. Nat Genet 2016; 48: 67–73. 26642243 10.1038/ng.3459 PMC4777523

[bib114] Boone DL, Turer EE, Lee EG, Ahmad RC, Wheeler MT, Tsui C et al. The ubiquitin-modifying enzyme A20 is required for termination of Toll-like receptor responses. Nat Immunol 2004; 5: 1052–1060. 15334086 10.1038/ni1110

[bib115] Turer EE, Tavares RM, Mortier E, Hitotsumatsu O, Advincula R, Lee B et al. Homeostatic MyD88-dependent signals cause lethal inflamMation in the absence of A20. J Exp Med 2008; 205: 451–464. 18268035 10.1084/jem.20071108 PMC2271029

[bib116] Onizawa M, Oshima S, Schulze-Topphoff U, Oses-Prieto JA, Lu T, Tavares R et al. The ubiquitin-modifying enzyme A20 restricts ubiquitination of the kinase RIPK3 and protects cells from necroptosis. Nat Immunol 2015; 16: 618–627. 25939025 10.1038/ni.3172 PMC4439357

[bib117] Vande Walle L, Van Opdenbosch N, Jacques P, Fossoul A, Verheugen E, Vogel P et al. Negative regulation of the NLRP3 inflammasome by A20 protects against arthritis. Nature 2014; 512: 69–73. 25043000 10.1038/nature13322 PMC4126806

[bib118] Duong BH, Onizawa M, Oses-Prieto JA, Advincula R, Burlingame A, Malynn BA et al. A20 restricts ubiquitination of pro-interleukin-1beta protein complexes and suppresses NLRP3 inflammasome activity. Immunity 2015; 42: 55–67. 25607459 10.1016/j.immuni.2014.12.031 PMC4302274

[bib119] Newton K, Dugger DL, Maltzman A, Greve JM, Hedehus M, Martin-McNulty B et al. RIPK3 deficiency or catalytically inactive RIPK1 provides greater benefit than MLKL deficiency in mouse models of inflammation and tissue injury. Cell Death Differ 2016; 23: 1565–1575. 27177019 10.1038/cdd.2016.46 PMC5072432

[bib120] Gonzalez-Juarbe N, Gilley RP, Hinojosa CA, Bradley KM, Kamei A, Gao G et al. Pore-forming toxins induce macrophage necroptosis during acute bacterial pneumonia. PLoS Pathog 2015; 11: e1005337. 26659062 10.1371/journal.ppat.1005337 PMC4676650

[bib121] Dondelinger Y, Jouan-Lanhouet S, Divert T, Theatre E, Bertin J, Gough PJ et al. NF-kappaB-independent role of IKKalpha/IKKbeta in preventing RIPK1 kinase-dependent apoptotic and necroptotic cell death during TNF signaling. Mol Cell 2015; 60: 63–76. 26344099 10.1016/j.molcel.2015.07.032

[bib122] Gunther C, Martini E, Wittkopf N, Amann K, Weigmann B, Neumann H et al. Caspase-8 regulates TNF-alpha-induced epithelial necroptosis and terminal ileitis. Nature 2011; 477: 335–339. 21921917 10.1038/nature10400 PMC3373730

[bib123] Weinlich R, Oberst A, Dillon CP, Janke LJ, Milasta S, Lukens JR et al. Protective roles for caspase-8 and cFLIP in adult homeostasis. Cell Rep 2013; 5: 340–348. 24095739 10.1016/j.celrep.2013.08.045 PMC3843376

[bib124] Takahashi N, Vereecke L, Bertrand MJ, Duprez L, Berger SB, Divert T et al. RIPK1 ensures intestinal homeostasis by protecting the epithelium against apoptosis. Nature 2014; 513: 95–99. 25186904 10.1038/nature13706

[bib125] Dannappel M, Vlantis K, Kumari S, Polykratis A, Kim C, Wachsmuth L et al. RIPK1 maintains epithelial homeostasis by inhibiting apoptosis and necroptosis. Nature 2014; 513: 90–94. 25132550 10.1038/nature13608 PMC4206266

[bib126] Newton K, Dugger DL, Wickliffe KE, Kapoor N, de Almagro MC, Vucic D et al. Activity of protein kinase RIPK3 determines whether cells die by necroptosis or apoptosis. Science 2014; 343: 1357–1360. 24557836 10.1126/science.1249361

[bib127] Piao X, Komazawa-Sakon S, Nishina T, Koike M, Piao JH, Ehlken H et al. c-FLIP maintains tissue homeostasis by preventing apoptosis and programmed necrosis. Sci Signal 2012; 5: ra93. 23250397 10.1126/scisignal.2003558 PMC4465294

[bib128] Zhang H, Zhou X, McQuade T, Li J, Chan FK, Zhang J. Functional complementation between FADD and RIP1 in embryos and lymphocytes. Nature 2011; 471: 373–376. 21368761 10.1038/nature09878 PMC3072026

[bib129] Welz PS, Wullaert A, Vlantis K, Kondylis V, Fernandez-Majada V, Ermolaeva M et al. FADD prevents RIP3-mediated epithelial cell necrosis and chronic intestinal inflammation. Nature 2011; 477: 330–334. 21804564 10.1038/nature10273

[bib130] Moulin M, Anderton H, Voss AK, Thomas T, Wong WW, Bankovacki A et al. IAPs limit activation of RIP kinases by TNF receptor 1 during development. EMBO J 2012; 31: 1679–1691. 22327219 10.1038/emboj.2012.18 PMC3321198

[bib131] Xanthoulea S, Pasparakis M, Kousteni S, Brakebusch C, Wallach D, Bauer J et al. Tumor necrosis factor (TNF) receptor shedding controls thresholds of innate immune activation that balance opposing TNF functions in infectious and inflammatory diseases. J Exp Med 2004; 200: 367–376. 15289505 10.1084/jem.20040435 PMC2211976

[bib132] Marsh RA, Madden L, Kitchen BJ, Mody R, McClimon B, Jordan MB et al. XIAP deficiency: a unique primary immunodeficiency best classified as X-linked familial hemophagocytic lymphohistiocytosis and not as X-linked lymphoproliferative disease. Blood 2010; 116: 1079–1082. 20489057 10.1182/blood-2010-01-256099 PMC2938130

[bib133] Mulay SR, Desai J, Kumar SV, Eberhard JN, Thomasova D, Romoli S et al. Cytotoxicity of crystals involves RIPK3-MLKL-mediated necroptosis. Nat Commun 2016; 7: 10274. 26817517 10.1038/ncomms10274 PMC4738349

[bib134] Kataoka K, Matsumoto H, Kaneko H, Notomi S, Takeuchi K, Sweigard JH et al. Macrophage- and RIP3-dependent inflammasome activation exacerbates retinal detachment-induced photoreceptor cell death. Cell Death Dis 2015; 6: e1731. 25906154 10.1038/cddis.2015.73 PMC4650542

[bib135] Qi X, Gurung P, Malireddi RK, Karmaus PW, Sharma D, Vogel P et al. Critical role of caspase-8-mediated IL-1 signaling in promoting Th2 responses during asthma pathogenesis. Mucosal Immunol 2016 doi:10.1038/mi.2016.1025. 10.1038/mi.2016.25 PMC5035164 27007676

[bib136] Wang X, Li Y, Liu S, Yu X, Li L, Shi C et al. Direct activation of RIP3/MLKL-dependent necrosis by herpes simplex virus 1 (HSV-1) protein ICP6 triggers host antiviral defense. Proc Natl Acad Sci USA 2014; 111: 15438–15443. 25316792 10.1073/pnas.1412767111 PMC4217423

[bib137] Nogusa S, Thapa RJ, Dillon CP, Liedmann S, Oguin TH 3rd, Ingram JP et al. RIPK3 activates parallel pathways of MLKL-driven necroptosis and FADD-mediated apoptosis to protect against influenza A virus. Cell Host Microbe 2016; 20: 13–24. 27321907 10.1016/j.chom.2016.05.011 PMC5026823

[bib138] Kitur K, Parker D, Nieto P, Ahn DS, Cohen TS, Chung S et al. Toxin-induced necroptosis is a major mechanism of staphylococcus aureus lung damage. PLoS Pathog 2015; 11: e1004820. 25880560 10.1371/journal.ppat.1004820 PMC4399879

